# Compacta: a fast contig clustering tool for de novo assembled transcriptomes

**DOI:** 10.1186/s12864-020-6528-x

**Published:** 2020-02-11

**Authors:** Fernando G. Razo-Mendivil, Octavio Martínez, Corina Hayano-Kanashiro

**Affiliations:** 1 Departamento de Investigaciones Científicas y Tecnológicas de la Universidad de Sonora, Universidad de Sonora, Hermosillo, Mexico; 2 Unidad de Genómica Avanzada (Langebio), Centro de Investigacíon y de Estudios Avanzados del Instituto Politécnico Nacional (Cinvestav), Irapuato, Gto Mexico

**Keywords:** RNA-Seq, de novo assembly, Corset, Grouper, Transcriptomics

## Abstract

**Background:**

RNA-Seq is the preferred method to explore transcriptomes and to estimate differential gene expression. When an organism has a well-characterized and annotated genome, reads obtained from RNA-Seq experiments can be directly mapped to that genome to estimate the number of transcripts present and relative expression levels of these transcripts. However, for unknown genomes, de novo assembly of RNA-Seq reads must be performed to generate a set of contigs that represents the transcriptome. These contig sets contain multiple transcripts, including immature mRNAs, spliced transcripts and allele variants, as well as products of close paralogs or gene families that can be difficult to distinguish. Thus, tools are needed to select a set of less redundant contigs to represent the transcriptome for downstream analyses. Here we describe the development of *Compacta* to produce contig sets from de novo assemblies.

**Results:**

*Compacta* is a fast and flexible computational tool that allows selection of a representative set of contigs from de novo assemblies. Using a graph-based algorithm, *Compacta* groups contigs into clusters based on the proportion of shared reads. The user can determine the minimum coverage of the contigs to be clustered, as well as a threshold for the proportion of shared reads in the clustered contigs, thus providing a dynamic range of transcriptome compression that can be adapted according to experimental aims. We compared the performance of *Compacta* against state of the art clustering algorithms on assemblies from Arabidopsis, mouse and mango, and found that *Compacta* yielded more rapid results and had competitive precision and recall ratios. We describe and demonstrate a pipeline to tailor *Compacta* parameters to specific experimental aims.

**Conclusions:**

*Compacta* is a fast and flexible algorithm for the determination of optimum contig sets that represent the transcriptome for downstream analyses.

## Background

RNA-Seq is the most frequently used method to explore transcriptomes, i.e., sets of mRNA molecules expressed in a cell, tissue, organ or whole organism under particular conditions [1, 2]. To generate samples for RNA-Seq, mRNA isolated from a given sample is converted to circular DNA (cDNA) that includes a mixture of fragments. The cDNA is sequenced to obtain ‘reads’ that represent parts of the original mRNA molecules. When a sample genome is known, the reads can be mapped to a reference sequence to reconstruct the transcripts and estimate their relative abundance.

However, when no genome is available, reads must be assembled de novo before attempting to reconstruct the expressed transcripts and estimate their relative abundance. Transcriptome assemblers including *Trinity* [3], *Soap* de novo [4], *ABySS* [5] or *Spades* [6], among others, perform this assembly to generate ‘contigs’ - sequences arising from reads that overlap or by the use of ‘Brujin graphs’ [7].

De novo assembly of eukaryotic transcriptomes is challenging both due to dataset size that can include billions of reads and the difficulties in identifying alternatively spliced variants [7], alternative gene alleles [8], small variants within a gene family [5] or close gene paralogs [9, 10]. This assembly problem is exacerbated by temporal transcription, wherein significant parts of the genome, both coding and non-coding segments, are transcribed only at specific points during development or under specific conditions [11, 12]. Moreover, a large fraction of reads can belong to nascent RNAs, and thus include introns that could contribute to many contigs in the assembly [13]. As a result, transcriptome assemblies typically produce very large contig sets that in some cases are many-fold larger than the number of genes in the entire species genome. For example, de novo assembly of the transcriptome for the polychaete annelid *Platynereis dumerilii* using *Trinity* gave a set of 273,087 non-redundant contigs, which were identified through a pipeline that included sequence homology to only 17,213 genes [14], nearly 16-fold fewer than the number of contigs.

Transcriptome assemblers output many contigs that reflect the diversity found in the original mixture of mRNA molecules. However, for downstream analyses, these large contig collections must be culled to yield a smaller and more tractable set, which ideally groups contigs into transcripts produced by the same gene. Methods to group contigs can involve the use of sequence information, such as *cd-hit-est* [15], or use only the information about which reads map to each contig. The two main programs using the second approach are *Corset* [16] and *Grouper* [17].

*Corset* takes the set of reads and hierarchically clusters the contigs based on the proportion of shared reads. The program first filters out contigs that have a low number of mapped reads (*<* 10 by default) and then cluster contigs based on shared reads, while separating contigs having different expression patterns between samples. This approach thus avoids placing two or more paralogs or alternatively spliced forms into the same cluster through the use of a likelihood ratio test across groups of samples having a fixed *P* value threshold of approximately 10^− 5^. A distance threshold for clustering can be set by the user, but the default value of 0.3 is equivalent to sharing of 70% of the reads between two entities, i.e., original contigs or clusters already obtained by the algorithm. The number of shared reads is also updated at each iteration and clustering of a contig set stops when either all the contigs have been grouped into a single cluster or the current minimum distance increases above the distance threshold.

The *Corset* algorithm has two disadvantages: First, it uses a fixed number of reads to assess contig coverage, disregarding contig and read lengths; Second, and perhaps more importantly, the *Corset* algorithm depends heavily on results of a likelihood ratio test to segregate into clusters those contigs that could be the product of two different genes. The nature and number of conditions used to obtain different transcriptome samples can be unpredictable and, in principle, extremely diverse. However, *Corset* output depends on these conditions and thus groups working with the same organism could conceivably obtain significantly different sets of clusters to represent the transcriptome. Also, for annotation of ongoing eukaryotic genome projects, an equimolar mixture of RNA from different tissues of the same species is sequenced [18]; in these cases the approach used by *Corset* that segregates contigs from the same gene is not useful because only one ‘condition’ is used and thus a maximum likelihood test cannot be performed.

*Grouper* is another algorithm that generates contig clusters based on shared reads. Similar to *Corset*, outputs generated by the *Grouper* algorithm exclude contigs having fewer than 10 reads; this threshold cannot be modified by the user. Also, like *Corset*, *Grouper* uses a likelihood ratio test of expression estimates that vary significantly across conditions to separate contigs under the assumption that such contigs arose from different paralogous genes. Optional *Grouper* filters allow information for ‘orphan’ reads (when paired reads are used), whereas the ‘min-cut’ filter uses the likelihood ratio test to completely separate contigs, thus avoiding long path joining. Interestingly, *Grouper* does not have a user adjustable threshold for weight (or distance) by which contigs are clustered and instead relies only on the abovementioned filters to cluster or segregate contigs. *Grouper* also has an associated module to label (annotate) clusters using information from a closely related genome.

*Grouper* shares the same disadvantages with *Corset*, i.e., the program uses an arbitrary minimum number of reads to consider whether a contig is valid (in *Grouper* the user cannot modify this value) and contig segregation depends on the RNA-Seq experimental conditions.

The ideal behavior for an algorithm to cluster contigs obtained by de novo assembly of a transcriptome would be to output a group of clusters (contig sets) that perfectly represent actual gene expression, i.e., a set wherein the relationship between cluster and gene is one to one. There are strong arguments concerning the impossibility of obtaining such an ‘ideal’ algorithm in the absence of detailed knowledge about the genome sequence in question and using only the information given by multi-mapping files that relate reads to contigs. In mathematical terms, we have an identifiability problem, meaning that different sets of parameters (genes) can give a set of reads having identical statistical profiles (number of reads per contigs), making it impossible to determine the set of genes that generated the output. As clearly demonstrated by [19], to correctly identify transcripts based entirely on RNA-Seq data, at minimum gene-boundary data are needed, and data concerning transcription start sites, splice junctions and polyadenylation sites are also useful. As noted by Boley et al. [19], “This means that it is not always possible to positively identify alternative transcript isoforms, even as the read depth approaches infinity”. Confronted with the problem of clustering contigs from an unknown genome, we have no information concerning factors such as genome size and complexity [20, 21], allele and gene copy variations [22] or variations in exon-intron architecture [23]. Under this scenario, the best use of information from de novo assembly is formation of a contig cluster that can be used to identify the core set of expressed genes that allows the most effective comparison of the relative expression of such entities based on the design of the RNA-Seq experiment.

With the aim of reducing the complexity of RNA-Seq data analyses, we present *Compacta*, a fast, flexible, and computationally efficient way to group contigs obtained from de novo assembly into clusters to represent the core set of genes expressed in a given experiment as well as to allow identification of gene sets and enhance statistical power for detection of differential expression. The algorithm depends on only two parameters: filtering of low coverage contigs based on effective coverage and clustering strength. After running *Compacta*, a single contig, representing each cluster obtained, can be used for downstream analyses for gene identification and detection of differential gene expression.

## Implementation

*Compacta* is designed to reduce the number of contigs to a smaller set of representative sequences while preserving the information about relative expression given by read abundance. Its output can be used for downstream analyses to identify contigs and differential gene expression patterns.

Prior to using *Compacta*, transcriptomes must be assembled de novo using tools such as *Trinity* [3], *Soap* de novo [4] or *Spades* [6]. Sequencing reads are then mapped back to the assembled transcriptome using alignment-based software such as *Bowtie2* [24] or *Hisat2* [25] to obtain a multi-mapped binary file in the ‘BAM’ format [26]. BAM files are the initial input for Compacta and contain information about the contig set given by the assembler as well as the reads that map to each set.

*Compacta* has two core parameters, −d = *d*, a threshold for when two contigs belong to the same cluster, and -l = *l*, the threshold needed for the minimum effective coverage for a contig to enter the clustering algorithm. The value for *d* ranges between zero and one and controls the extent of clustering. When *d* = 0*.*3, for example, all pairs of contigs sharing 30% or more of the reads that reference the contig having fewer reads will be clustered into a single entity. Meanwhile, *l* = 2 implies that only those contigs having a total coverage that is twice the contig length in terms of sequencing read lengths will enter into the clustering process. Default values for these two parameters are *d* = 0*.*3 and *l* = 2, which are determined in the input as “-d 0.3 -l 2”. In addition to file locations, *Compacta* includes options for number and names of samples and experimental groups, as well as options that allow parallelization of part of the algorithm.

*Compacta* output comprises files that: (i) define the obtained clusters as sets of the original contigs; (ii) give the number of reads (raw count) of each cluster for each sample input; and (iii) describe the type of clusters obtained. The following list describes the parameters of the Compacta algorithm.

1. **Input.** A set of BAM files and *Compacta* options. BAM file data are parsed for the next step. The sample origin of reads is preserved for inclusion in the output.
2. **Graph computation.** From sets of *c* contigs and *r* reads in BAM files, *Compacta* creates an undirected graph with *c* vertices corresponding to contigs and *c*(*c* − 1)*/*2 connections (edges) between vertices. The weight, *w*_*ij*_, of an edge connecting contigs *i* and *j*; *i* ^*−*^ *j*, is calculated
  $$ {w}_{ij}=\frac{R_{ij}}{\mathit{\min}\left({R}_j\right)} $$

where *R*_*i*_ and *R*_*j*_ are the number of reads that independently map to contigs *i* and *j*, respectively, while *R*_*ij*_ is the total number of reads that map to both contigs *i* and *j*; i.e., *R*_*ij*_ is the number of reads shared by contigs *i* and *j*. This function is well defined since min (*R*_*i*_*,R*_*j*_) *>* 0. The weight of an edge, *w*_*ij*_, ranges from zero, when the edge contigs share no sequencing reads indicating no similarity (disconnected contigs), to one, indicating that one of the contigs is a proper subset of the other.

1. **Filtering of low evidence contigs.** The value *c*_*i*_ is defined as the length of contig *i* and *s*_*i*_ is the sum of the lengths of all reads that map to that contig. If *s*_*i*_ *<* (*l* × *c*_*i*_), where *l* is the parameter ‘-l’ input by the user, the contig *i* is disconnected from any other vertices in the graph and will be reported as a ‘low evidence contig’. Disconnection of contig *i* implies setting all weights *w*_*ij*_ = 0 for all values of *j*, in turn implying that when the set of contigs considered in subsequent algorithm steps fulfill the condition *s*_*i*_ ≥ (*l* × *c*_*i*_), they are considered to be contigs with sufficient evidence of expression.
2. **Pre-cluster detection.** Connected contigs (vertices) are detected and isolated sub-graphs are marked as ‘pre-clusters’ that are each loaded into a heap structure self-ordered by edge weight, ensuring that the first value in the heap is always the edge having the heaviest weight, i.e., the largest value of *w*_*ij*_.
3. **Clustering.** *Compacta* processes each pre-cluster using an agglomerative algorithm. At each iteration, the algorithm selects the edge having the highest weight and, if this weight is above the defined threshold *d* (parameter input as -d), the nodes are grouped into a new entity. In this scenario, weights, *w*_*ij*_, are re-calculated for the new conformation of the pre-cluster and the process is repeated until the first edge in the heap has a weight that is less than the threshold *d* or all its contigs are clustered together. The final content of the heap structure, which can contain one or more clusters, goes to the output.
4. **Output.** Once *Compacta* processes all pre-clusters, it produces files that include the description of each cluster (sets of the original contigs), as well as lists indicating which contig could represent each one of the clusters, either by being the longest contig in the cluster or the one that has the largest number of reads mapping to it.

In summary, from BAM files containing the information of the original contigs and reads mapping to them, *Compacta* produces a set of representative contigs for use in downstream analyses.

### Algorithm implications

As with other software designed to reduce transcriptome complexity, such as *Corset* or *Grouper*, *Compacta* uses a graphical approach that ignores nucleotide sequence and considers contigs only as sets of sequencing reads. Two contigs, *i* and *j*, will be connected in the graph if they share some reads, i.e., if their intersection is not empty and *w*_*ij*_ *>* 0. In step (2) of the algorithm, the graph is constructed. Even when in principle all pair comparisons between contigs must be performed, only the ones for which the weights are larger than zero (*w*_*ij*_ *>* 0) need to be stored and analyzed downstream. The logic behind weight calculation is that contigs sharing a large proportion of reads will also be ‘alike’ at the sequence level, allowing read position within contigs to be disregarded. Thus, if *w*_*ij*_ = 0 we will consider that the corresponding contigs are completely unrelated, whereas *w*_*ij*_ = 1 means that the smaller contig is a proper subset of the second, or, when they are the same size, they will be some permutation of the positions of the same reads.

In step (3) of the algorithm, *Compacta* uses effective contig coverage, expressed as the number of times that the full-length contig is covered by reads, as a measure to detect and discard low evidence contigs. The user controls the strength of filtering via parameter *l*; By setting *l* = 3, for example, only those contigs having sufficient numbers of reads to cover the contig length three times will pass the filter and continue for downstream analysis. This parameter allows the user to limit the subset of contigs of interest. Thus, if only those genes having high expression levels are relevant, *l* can be set to a high value. Filtered contigs are not discarded, but are included in the output in which they are identified as ‘low evidence singletons’. In contrast, *Corset* and *Grouper* allow selection of contigs only through a fixed threshold in the number of reads that map to each contig, independently of contig length. In *Corset* this threshold can be changed by the user and by default is set to 10, while in *Grouper* the threshold is fixed as 10 reads. However, a fixed threshold number of reads is inadequate to judge contigs having different lengths. For example, consider the situation in which reads of 250 bp are used and a contig of length 750 bp is produced by 9 overlapping reads. Here, the effective contig coverage is (250 × 9)*/*750 = 3, and *Compacta* will reasonably pass such a highly covered contig for any value of *l* ≤ 3, whereas *Corset* and *Grouper* would discard such a contig considering it as ‘low coverage’, and thus it would not appear in the output.

The graph constituted by all contig pairs having *w*_*ij*_ *>* 0 are input into the fourth step of the algorithm, ‘pre-cluster detection’. Here a pre-cluster is defined as a set of inter-connected contigs, or, in graph theory terms, as a ‘connected graph’ [27]. In simple terms, in a pre-cluster there is a path that connects, either directly or indirectly, all contigs that form such a structure. If a pre-cluster graph is plotted, it is possible to go from any of the contigs to any other contig by following a path. An important computational advantage of *Compacta* is that each pre-cluster is loaded into a self-ordered heap structure, in which the first edge always has the largest *w*_*ij*_ value. This heap structure is similar to ordered binary trees, and can save considerable time [28], because arrays having millions of components are not sorted at each iteration.

The core of the *Compacta* algorithm is step (5), involving agglomerative clustering of connected contigs or ‘pre-clusters’ that can be performed in parallel. The processing of each pre-cluster is independent of other data, and thus its clustering can be sent as an independent thread, making optimal use of computer resources. With the same goal, sets of pre-clusters could be distributed to independent nodes in computer clusters. Clustering of a pre-cluster structure proceeds by grouping into a single entity pairs of sets having weight *w*_*ij*_ that surpass the threshold *d* input by the user. Given that the pre-cluster is loaded into a self-ordered heap, the algorithm needs only to analyze the first element of the heap, thus saving valuable time. Clustering of two entities, *i* and *j* (that could be original contigs or previously identified clusters), happens only if *w*_*ij*_ ≥ *d* and in that case both entities are grouped together, after which weights between the new entity and all those in the pre-cluster are re-calculated and the algorithm iterated. In the opposite case, such as when *w*_*ij*_ *< d* during the iterations, the entire content of the heap is sent to the output, including the definitions of clusters and the number of reads that map to them. This process guarantees that the number of entities in the output is smaller than or at most equal to the number of input contigs. A simple example of this process is presented in Section 1 of Additional file 1.

Any contig clustering algorithm that does not use direct sequence information but instead uses a graphical approach must have a parameter homolog to the weight threshold *d* used by *Compacta*. For example, in *Corset* and *Grouper* this homolog parameter is the distance between contigs, which is simply the inverse additive of *Compacta d*, i.e., 1 − *d* for the threshold and 1 − *w*_*ij*_ for the weights, which in these programs are conceptualized as distances. In addition to the criterion used to filter ‘low evidence contigs’ as mentioned earlier, computational implementation of *Compacta* differs from those in *Corset* and *Grouper* in the use of efficient self-sorting heap structures to dynamically store pre-clusters, which in turn allows the clustering step of *Compacta* to be fully parallelized or distributed, thus making optimum use of computer resources, including multi-core clusters.

Another substantial way that *Compacta* differs from *Corset* and *Grouper* is that *Compacta* uses no computational methods to determine if two contigs were the product of transcription from ‘the same gene’, whereas both *Corset* and *Grouper* attempt to estimate and consider contig origin. In our opinion, in the absence of genomic information, accurate prediction of whether two contigs are the product of: a) different alleles of the same gene, b) alternative splicing forms produced from the same gene or c) two highly similar genes (close paralogs or two close members of the same gene family) is essentially impossible due to the high diversity and conformations of eukaryotic genomes.

*Compacta* will be particularly useful when no genome is available for a given organism, and the researcher wants to: a) Have a core set of sequences representing the major expressed genes that allows putative identification via comparisons with well-known orthologs; and b) Perform differential expression analysis of core genes expressed in the transcriptome. To achieve these aims, the ability to downsize the potentially very large number of contigs given by the assembler into a smaller and more manageable set of representative sequences is valuable.

### Adjusting *Compacta* to assembly complexity

RNA-Seq experiments capture many transcript types such as nascent or pre-mature RNAs [13] or non-coding sequences like long non-coding RNAs [29]. In fact, the ratio of transcribed non-coding to coding sequences can vary enormously; in humans this ratio is 47:1, but in nematodes is only 1.3:1 [30]. The assembly process is likely to yield many related contigs that represent transcription variants of the same gene as alternative splicing forms, alleles, or products of the transcription of close paralogs of the same gene or gene family. Here we discuss the features that Compacta offers to reduce assembly complexity in a general framework.

Given a particular assembly, say **t**, consisting of a group of *c* contigs and *r* reads related by multi-mapping files (‘BAM’ files), we can use *Compacta* to reduce the set of c contigs to a smaller set of *z* representative clusters such that *z* ≤ *c*. Apart from filtering low-evidence contigs with the parameter -l = *l*, the number of clusters given by the algorithm is a function only of the parameter *d* –the threshold for clustering contigs into clusters, say *f*(**t***,d*) = *z*, or simply *f*(*c,d*) = *z*, considering only the number of input contigs, *c*, and the number of clusters output, *z*. By setting *d* = 0 we will cluster all contigs that share one or more reads, because in that case all contig pairs {*i,j*} that fulfill *R*_*ij*_ *>* 0 will give a weight *w*_*ij*_ *>* 0 and thus be clustered together, giving the smallest number of clusters in the output. The number of clusters resulting from that operation can be termed *z*_*min*_, where *f*(*c,d* = 0) = *z*_*min*_, which represents the maximum assembly reduction that can be achieved by the algorithm. By clustering all contigs with the slightest evidence of sequence similarity (i.e., one or more shared reads) we can group all alleles, alternative splicing variants and close paralogs genes into a single cluster. However, using this approach we could also group into a single cluster transcripts produced by different genes that share sequence motifs that expand in sequence length beyond the length of a single read. Under the same experimental conditions, and with high sequencing depth, we can assume that read length will have a strong effect in determining the value of *z*_*min*_; short reads will cause *z*_*min*_ to be smaller than when long reads are used. On the other hand, if *d* is set to 1, we will ask the algorithm to group only contigs that share all reads of the smaller contig, because in order to have *w*_*ij*_ = *R*_*ij*_*/*min (*R*_*i*_*,R*_*j*_) = 1 we must have *R*_*ij*_ = *R*_*i*_ or *R*_*ij*_ = *R*_*j*_. In that case, we will have a maximum number of clusters in the output, where *f*(*c,d* = 1) = *z*_*max*_, such that *Compacta* will cluster only those contigs that are proper subsets of the longest contig in the group (pre-cluster) and will likely produce clusters containing only highly similar gene alleles, splicing forms that share most exons in the genes, or very close paralogs. Taken together, from this analysis we can conclude that *f*(*c,d*) = *z* is a non-decreasing function of *d* with domain in the interval [0*,*1] for *d* and co-domain in [*z*_*min*_*,z*_*max*_] for *z*. The fact that *f*(*c,d*) = *z* is non-decreasing follows from the fact that a larger value of *d* can only increase the number of output clusters, *z*, given that the clustering algorithm will be more stringent, i.e., if *d*_1_ *< d*_2_ then *f*(*c,d*_1_) ≤ *f*(*c,d*_2_). Due to the speed of *Compacta*, performing two runs with extreme values, *d* = 0 and *d* = 1, to obtain the values of *z*_*min*_ and *z*_*max*_ for a particular assembly is not computationally expensive. Having the range of possible *z* values allows the researcher to fix a target value *z*^∗^, *z*_*min*_ ≤ *z*^∗^ ≤ *z*_*max*_, and, using a numerical method, obtain the value of *d* (e.g., *d*^∗^), such that *f*(*c,d*^∗^) ≈ *z*^∗^ by performing a set of Compacta runs.

### Source data and software evaluation

Three RNA-Seq datasets from Arabidopsis (*Arabidopsis thaliana*), mango (*Mangifera indica*) and mouse (*Mus musculus*) were processed to compare *Compacta* with other clustering tools.

In Table 1 the ‘Source’ column provides the reference for the corresponding dataset; the column ‘Accession’ shows accession identifiers for data deposited in the Sequence Read Archive [34] of GenBank; the column ‘Reads (Gb)’ indicates the approximate giga base pairs of raw data; and ‘Contigs’ shows the number of contigs obtained from the assembly. The Arabidopsis and mouse datasets were assembled de novo using the *Trinity* assembler version 2.4.0 with default parameters, whereas the mango dataset assembly generated by *Trinity* was kindly provided by Dr. Miguel A. Hernández Oñate [32].

**Table 1:** Data sources. Sources and characteristics of the RNA-Seq data used in this study

Organism	Source	Accession	Reads (Gb)	Contigs
Arabidopsis	[31]	ERP016911	36.0	106,895
Mango	[32]	SRP043494	62.5	107,744
Mouse	[33]	PRJNA474181	41.0	327,616

**Fig. 1:**
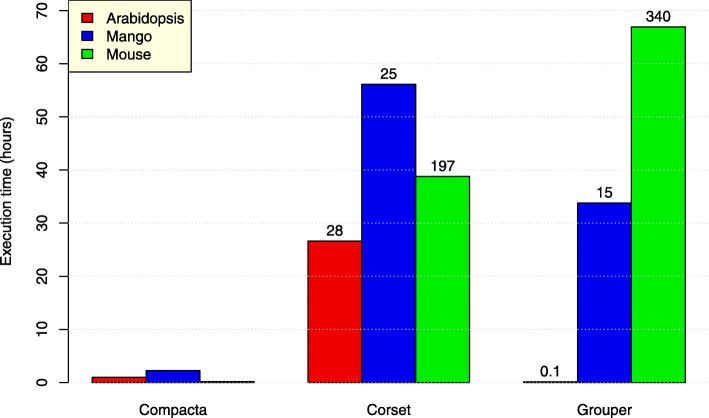
Execution time for *Compacta, Corset* and *Grouper* in three assemblies. Bar diagram of running time in hours for *Compacta*, *Corset* and *Grouper* algorithms to analyze assemblies from Arabidopsis, mango and mouse. Numbers in the upper bars for *Corset* and *Grouper* are the number of rounds that the execution took for the corresponding program compared with the *Compacta* execution time

*Compacta*, *Corset* and *Grouper* were run with default parameters using as input the contigs for each assembly obtained from the sources shown in Table 1 (Fig. 1).

**Fig. 2:**
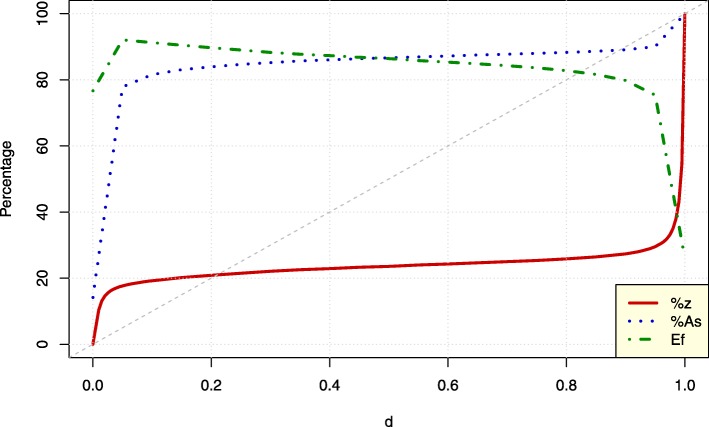
*Compacta* results for the Arabidopsis assembly. Values for *d* are displayed on the *X*-axis and the *Y*-axis shows the percentage of clusters (*z*; red line), number of Arabidopsis sequences identified (*n*_*As*_; blue dotted line) and efficiency (*Ef = n*_*As*_*/z*; green dashed line) as a function of *d*

Results shown in Fig. 2 were obtained using Arabidopsis assembly contigs (see Table 1) and performing repeated runs of *Compacta* using different values of the *d* parameter, whereas all contigs from such assemblies were identified by comparing those sequences using stringent *BLAST* parameters [35] with the set of all possible Arabidopsis transcripts. Details of this analysis are given in Section 3 of Additional file 1.

**Fig. 3:**
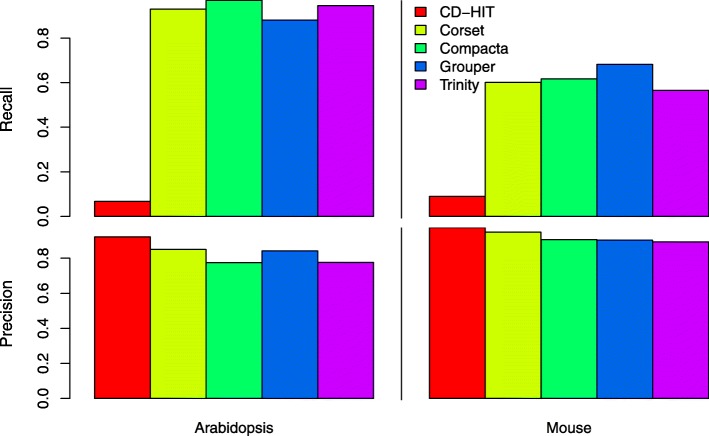
Estimated Recall and Precision of 5 programs in two assemblies. Bar plots for Recall (upper) and Precision (lower) of 5 de novo assembly clustering algorithms applied to two assemblies, Arabidopsis (left) and mouse (right)

Results presented in Fig. 3 were obtained by running *CD-HIT*, *Compacta*, *Corset*, *Grouper* and the clustering facility of the *Trinity* suite on the contigs from assemblies of the Arabidopsis and mouse datasets (Table 1); details of these experiments as well as additional analyses are given in Sections 2 and 3 of Additional file 1.

## Results and discussion

### *Compacta* is faster than clustering alternatives

To evaluate the absolute and relative execution time for *Compacta*, *Corset* and *Grouper* we used three transcriptomes from Arabidopsis, mango (*Mangifera indica*) and mouse (*Mus musculus*) assembled de novo that included 106,895, 107,744 and 327,616 contigs, respectively. All three algorithms were run with default parameters and the run time for each program with each assembly was obtained (Fig. 1; see Material and Methods for details). Table 2 shows the number of clusters output by *Compacta*, *Corset* and *Grouper* for the Arabidopsis, mouse and mango datasets. *Compacta* produced a larger number of contigs in the Arabidopsis and mouse real datasets, and the smaller number of contigs for the mango dataset and the simulated datasets of Arabidopsis and mouse. This reflects the fact that *Corset* and *Grouper* do not include contigs with low coverage in their output, while *Compacta* includes contigs with low coverage as single contig clusters.

**Table 2:** Number of representative contigs selected by each algorithm from each transcriptome when run with default parameters

	Arabidopsis		Mouse		Mango
	Real	Simulated	Real	Simulated	Real
Compacta	33,542	21,518	223,169	19,844	28,356
Corset	27,080	26,414	95,079	23,716	38,448
Grouper	27,949	23,026	57,501	18,652	38,063

In Fig. 1 the bar height corresponds to the run time for each program (bar group; *X*-axis) operating on the three assemblies that are denoted by different colors. The numbers above the bars for “Corset” and “Grouper” groups give the time taken by the program divided by the time taken by *Compacta* to analyze the same assembly. For example, the number 28 above the red bar for the “Corset” group indicates that *Corset* took approximately 28-fold more time to finish the run for the Arabidopsis assembly than *Compacta* (26*.*6186 *h/*0*.*9675 *h* ≈ 28).

*Compacta* was approximately 28-, 25- and 197-fold faster than *Corset* for the Arabidopsis, mango and mouse assemblies, respectively. The differences in execution time could be attributed to two factors: First, *Corset* uses a statistical formula to try to evaluate the gene of origin for each contig and *Compacta* does not; and Second, *Compacta* uses auto-sorting heaps, whereas *Corset* sorts all remaining contigs pairs in each iteration. A basic agglomerative clustering algorithm, such as that implemented for *Corset*, has a computation time of *O*(*n*^3^) and slows as the input size increases, as demonstrated by [28]. As mentioned above, *Compacta* uses an agglomerative algorithm with a heap that auto-sorts elements upon insertion and deletion that reduces computation time up to *O*(*n*^2^ log*n*) [28], which is considerably faster than the other algorithms, particularly when the size of the input data increases. Although *Compacta* may not always be faster than *Corset* for all possible assemblies, we predict that *Compacta* will be at least 10 times faster than *Corset* for any complex assembly from eukaryotic organisms. This prediction is based not only on our experimental results (Fig. 1), but also in the fundamentally more efficient way in which *Compacta* handles contig clustering by avoiding sorting the pre-cluster structure at each iteration, which adds significantly to the *Corset* run time.

On the other hand, in comparing *Grouper* and *Compacta* we see that *Compacta* is faster than *Grouper* for the mango and mouse assemblies by 15- and 340-fold, respectively, but slower for the Arabidopsis assembly for which *Compacta* took 0.9675 h and Grouper took only 0.1332 h, a ratio of ≈ 0*.*1 in favor of *Grouper*. The difference seen between *Grouper* and *Compacta* in processing the Arabidopsis assembly is due to *Grouper*’s use of equivalence files, which are simpler to parse and contain less information than the BAM files used by *Compacta*. However, for larger and more complex assemblies, such as those for mango and mouse, input file parsing represents a much small fraction of the total processing time, such that *Compacta* is faster than *Grouper* (c.f., *Compacta* was 340-fold faster than *Grouper* for the mouse assembly; last bar in Fig. 1). Moreover, *Grouper* relies on a Stoer–Wagner min-cut function that is repeatedly executed with a computation time of *O* (*mn* + *n*^2^ log*n*) [36], whereas *Compacta* uses the auto-sorting heap that has an execution time of *O*(*n*^2^ log*n*) [28]. Conservatively, *Compacta* is at least 10-fold faster than *Grouper* for clustering complex eukaryotic assemblies.

### *Compacta* adaptability to RNA-Seq objectives

As shown above, *Compacta* can be adjusted through the *d* parameter to give a number of clusters within the range [*z*_*min*_*,z*_*max*_], where *z*_*min*_ corresponds to *d* = 0 and *z*_*max*_ corresponds to *d* = 1. Here we demonstrate and discuss the consequences of selecting particular values for *d* to adjust the clustering results according to the particular aims of an RNA-Seq experiment. Because *Compacta* is fast, runs can be performed for a grid of *d* values and the results can be used for downstream analyses to identify genes and detect differential expression. Compared with the costs of RNA-Seq library construction and sequencing, the costs for bioinformatic analyses are negligible such that more time can be spent to perform such analyses to obtain optimum information from the data. Each *d* value gives *z* clusters, and the researcher can then select *z* contigs (one for each cluster) and use tools such as *BLAST* [35] to identify representative contigs in the transcriptome of a related species, or *BUSCO* [37] to assess contig set completeness. Test runs of differential expression programs, such as *edgeR* [38], can be performed with each representative set of contigs to evaluate the suitability of the results for achieving the particular aims of an RNA-Seq experiment. Here we show results obtained by running *Compacta* with the Arabidopsis assembly that generated the speed test results (Fig. 1; see Source data and Software evaluation and Section 3 of Additional file 1 for details).

The Arabidopsis assembly resulted in 106,895 contigs that were sent as a query to a *BLAST* database containing the full Arabidopsis cDNA set, which comprises 41,671 different sequences. *BLAST* hits were filtered by coverture, bit score and E-value giving a total of 23,607 significant concordances. The Arabidopsis assembly was run through *Compacta* using a grid of *d* values and with the parameter *l* set to its default value of 2 (for details see Section 3 of Additional file 1).

Table 3 shows that the dynamic range of *Compacta* for this assembly goes from *z*_*min*_ = 13,770 clusters when *d* = 0 to *z*_*max*_ = 103,262 clusters when *d* = 1; a 7.5-fold change between the maximum and minimum values. By taking the largest contig as representative of each cluster, the number of distinct Arabidopsis sequences identified (column *n*_*As*_ in Table 3) varies from a minimum of 3344 when *d* = 0 to a maximum of 23,607 for *d* = 1. This latter value corresponds to the total number of Arabidopsis sequences identified in the entire assembly. The ratio between the maximum and minimum of Arabidopsis sequences identified is 23,607 / 3344 ≈ 7, which is similar to the ratio *z*_*max*_*/z*_*min*_ of ≈ 7.5 However, we will see that the proportion of changes in the number of clusters and identified sequences do not follow a linear function of *d*.

**Table 3:** *Compacta* results for the Arabidopsis assembly. *d* - Parameter value, *z* - Number of clusters (representative contigs), *n*_*As*_ - Number of Arabidopsis sequences identified

*d*		*z*	*n*_*AS*_
0.000		13,770	3344
0.035	*(d*_*l*_*)*	28,704	18,381
0.500		34,860	20,455
0.955	*(d*_*r*_*)*	40,656	21,254
1.000		103,262	23,607

The percentages of clusters, *z*, number of identified sequences, *n*_*As*_, and clustering efficiency, *Ef*, defined as *Ef* = *n*_*As*_*/z*, are estimated as curve functions of *d*, which again does not have a linear relationship as shown by the grey dashed line with slope 10 (Fig. 2). Nevertheless, these three curvilinear functions show a relatively small slope (≈ 0) for *d* values between 0.1 and 0.9, whereas in the left and right hand extremes the three functions show sudden slope changes; from very high to relatively low near *d* = 0*.*035 and, conversely, from a relatively low to a very high slope near *d* = 0*.*955 (Fig. 2; see Table 3 for the estimated values at these critical points). Although generalizations cannot be made based on this one example, it is reasonable to assume that for almost all assemblies the values for number of clusters, identified sequences and efficiency will be non-linear functions of *d* having two critical points at which a sudden slope change at *d*_*l*_ ≈ 0 and *d*_*r*_ ≈ 1 and a relatively flat change (low slope) around *d* = 0*.*5 occur. The values for *d*_*l*_ and *d*_*r*_ are shown in Table 3 for the Arabidopsis assembly, and these values can be easily estimated for any particular assembly.

The curves for the Arabidopsis assembly show that there will be an unavoidable loss of accuracy in the analysis of assemblies obtained de novo from RNA-Seq experiments (Fig. 2). This inaccuracy results from not taking into account genome architecture, which in turn can confound identification of the gene from which each transcript originated to create multiple putative transcripts of which an unknown proportion could be artifacts, i.e., transcripts that do not exist physiologically. Fortunately, clustering assemblies to reduce this complexity can address the problem.

In the following discussion we will assume that the researcher has putatively identified all original contigs, has determined the approximate points *d*_*l*_*, d*_*r*_ for the assembly of interest, has obtained sets of representative contigs at *d* equal to 0*, d*_*l*_*,* 0*.*5*, d*_*r*_ and 1, and performed differential expression analyses for all *d* values shown above. We will be looking for a ‘Goldilocks’ point for *d* where there are not ‘too few’ or ‘too many’ contigs to obtain biologically relevant knowledge from our data.

Analyses of a representative set of contigs at *d* = 0 will give the minimum resolution of the assembly, because all representative contigs at that point represent completely independent ‘genes’ or ‘gene families’ and consequently the number of identified sequences will be a minimum (Fig. 2; blue dotted line). Based on this selection, all reads mapping to the contigs will have a unique hit, and thus the statistical power of detection of differentially expressed entities will be a maximum, because reads are not shared between the entities analyzed. Aside from other considerations, the differential expression analysis at *d* = 0 has the advantage of displaying a broad scenario; entities that are differentially expressed will show sets of genes that are surely affected by experimental conditions, even if many splicing variants and other similar transcripts are grouped and represented by a single transcript.

However, for many purposes, the number of representative sequences at *d* = 0 will be ‘too few’ and further analyses will be needed to improve accuracy.

On the other extreme, at *d* = 1, we have the largest number of representative contigs, and consequently the largest number of identified sequences (Fig. 2; dotted blue line). However, the procedure efficiency is lowest at this point (Fig. 2; dashed green line), meaning that many contigs represent the same identified sequence (*Ef* = *n*_*As*_*/z* is at its minimum), and thus very little advantage is gained from the clustering procedure, because many representative sequences will be redundant and characterize the same gene. At this point, the statistical power is also reduced because fewer reads map to each individual contig when compared with the other extreme (i.e., *d* = 0). Thus, *d* = 1 will give us ‘too many’ contigs.

Examining the point around which *d* = *d*_*l*_ for the Arabidopsis assembly gives *d* ≈ 0*.*035 (Table 3). At approximately this point the curves for *z,n*_*As*_ and *Ef* show a sudden slope change, going from a sharp increase to a more steady state that will continue for values of *d > d*_*l*_ and up to *d < d*_*r*_. The *d*_*l*_ point gives large increases in the number of contigs, *z*, and identified sequences, *n*_*As*_, when compared with the point at *d* = 0, say ∆*z*(*d*) = *z*(*d* = *d*_*l*_) − *z*(*d* = 0) = 28,704–13,770 = 14,934 (≈ 15% of increment), ∆*n*_*As*_(*d*) = *n*_*As*_(*d* = *d*_*l*_) − *n*_*As*_(*d* = 0) = 18,381–3344 = 15,037 (≈ 65% of increment; see Table 3 and Fig. 2). Also near *d* = *d*_*l*_, at *d* = 0*.*05, we obtain the maximum efficiency, max (*Ef*) = 18,381*/*19962 ≈ 0*.*92 (see Fig. 2 and Section 3 of Additional file 1 for details). In the context of information content, the d value at which max (*Ef*) is reached is optimal; i.e., we will not have ‘too few’ or ‘too many’ contigs to represent assembly diversity and thus this point is the ‘Goldilocks point on *d*’. If the differential expression of the representative contigs at max (*Ef*) is satisfactory for the aims of the RNA-Seq experiment, the analysis at this point could be reported as the final result.

The pipeline sketched here to obtain an optimum point for the parameter *d* that corresponds to the maximum efficiency, max (*Ef*), can be easily performed with an assembly obtained from any RNA-Seq experiment. In general, the most complex decision for the researcher is selection of a well-known and closest organism with which to suitably compare the organism under study. When such selection is done, a simple *BLAST* experiment using all contigs from the assembly as queries and the full transcriptome of the known organism as the target can be easily performed (details of this procedure are presented in Section 3 of Additional file 1 for the Arabidopsis assembly). Alternatively, or additionally, the researcher could perform a BUSCO experiment (see [35] and the website with [37]). The general lines of the procedure are, first, to obtain all *BUSCO* terms that correspond to all contigs in the assembly, and upon gathering these terms, use the sets of representative contigs obtained with *Compacta* with a grid of *d* values. As with *BLAST*, with *BUSCO* we can obtain a point *d* that will correspond to the maximum efficiency, max (*Ef*), but in this case the *Ef* for each value of *d* is defined as the number of *BUSCO* terms found over the number of contigs. An additional advantage of the *BUSCO* approach is that the terms found will generally have straightforward biological interpretations, which is useful for understanding differential expression analyses.

### Comparing *Compacta* with other clustering tools

*Compacta* does not directly use sequence information (as do tools like *CD-HIT* [15]) but instead uses reads that are shared between contigs. It also does not use statistical approaches to try to determine the gene of origin for each contig (as do *Corset* or *Grouper*), and as such is not fully comparable with all clustering tools. However, we did perform various comparisons and the results are summarized below, with details and other analyses presented in Section 2 of Additional file 1.

An ideal clustering algorithm for assemblies will correctly identify all contigs that arise from transcription of the same locus and cluster all of these contigs together. On the other hand, contigs arising from transcripts originating from different loci will always belong to different clusters. However, two kinds of errors, false positives (where two contigs from different loci are clustered together) and false negatives (where two contigs that belong to the same locus are not clustered together) can occur.

In Table 4 we present four possibilities for the classification of contig pairs, and the frequency of each one of the cases (in each of the 4 cells of the table) is represented by *a, b, c* and *d*, whereas the sum of the frequencies, *a* + *b* + *c* + *d*, gives the total number of pair-wise contig comparisons. A perfect clustering algorithm will have *b* = 0 and *c* = 0, and we can define two metrics to measure an algorithm behavior, termed ‘Recall’, *R* = *a/*(*a* + *c*) and ‘Precision’, *P* = *a/*(*a* + *b*). Clearly, the perfect algorithm will have *R* = 1 and *P* = 1, whereas *R <* 1 imply the existence of false negatives (*c >* 0) and *P <* 1 implies the existence of false positives (*b >* 0).

**Table 4:** Case classification for contig pairs after clustering. *a, b, c* and *d* are frequencies resulting from a clustering experiment

		Same locus?		
		Yes	No	Total
Clustered?	Yes	*a* (true positive)	*b* (false positive)	*a* + *b* (positives)
	No	*c* (false negative)	*d* (true negative)	*c* + *d* (negatives)
	Total	*a* + *c* (single locus)	*b* + *d* (different loci)	*a* + *b* + *c* + *d*

We estimated recall, *R*, and precision, *P*, of 5 clustering algorithms for Arabidopsis and mouse assemblies that were used to evaluate execution time (see Fig. 1 and Source data and Software evaluation). We again compared *Compacta* with mainly graph-based *Corset* and *Grouper* and also with *CD-HIT*, which relies exclusively on sequence information [15] and the *Trinity* program for contig grouping [39], which is based on De Bruijn graphs. For the comparison, all five programs were run using default parameters.

The program having the lowest recall for both assemblies was *CD-HIT*, and the values significantly differed from the higher values produced by the other 4 programs (Fig. 3). This difference could be because *CD-HIT* uses only information for direct contig likeness at the sequence level, and thus produces multiple false negatives when contigs that are truly related are not grouped. On the other hand, *CD-HIT* had the highest precision for both assemblies, implying a low frequency of false positives that again is attributable to the direct use of sequence information: when *CD-HIT* clusters two or more contigs, it does so based on high sequence similarity. A disadvantage of *CD-HIT* is that the clusters it produces contain only a small number of contigs –those that are highly similar at the sequence level, and the user has little control over the degree of assembly compression (data not shown; see Section 2 of Additional file 1).

We also observed that the recall ratios for *Corset*, *Compacta*, *Grouper* and *Trinity* were relatively similar within both mouse and Arabidopsis assemblies, but the values for mouse were always lower than those for Arabidopsis (Fig. 3). The higher complexity of the mouse assembly relative to that for Arabidopsis can explain the generally lower recall ratios of the four programs and that higher numbers of false negatives can be produced with complex assemblies. *Compacta* had the highest recall for Arabidopsis, while *Grouper* had the highest recall for the mouse assembly.

In contrast to the results for recall, for all five programs precision was marginally higher for the mouse assembly relative to that for Arabidopsis (Fig. 3), suggesting that the relative proportion of false positives does not increase with assembly complexity.

In terms of precision, and excluding the *CD-HIT* case discussed above, in all cases *Corset*, *Compacta*, *Grouper* and *Trinity* have a precision around 0.8 (Fig. 3); at *P* = 0*.*85 and *P* ≈ 0*.*84 *Corset* and *Grouper*, respectively, had the highest precision for the Arabidopsis assembly, whereas *Compacta* and *Trinity* both had *P* ≈ 0*.*78. For the mouse assembly, *Corset* had the highest precision (*P* ≈ 0*.*95) while *Compacta* and *Grouper* both had *P* ≈ 0*.*9, and *Trinity* had *P* ≈ 0*.*89. Even when *Compacta* did not have the highest precision in the assemblies studied, it was faster than the other programs, and, importantly, can be adjusted to yield an optimum number of clusters with the highest efficiency (see previous section). These advantages compensate for the minor loss of precision upon comparing Compacta with Corset and Grouper. Furthermore, because Compacta does not try to determine the gene origin of each contig, its results are independent of the RNA-Seq experimental design (treatments), whereas Corset and Grouper are affected by experimental factors due to the use of statistical tools to try to estimate genes from which contigs originated.

We compared the precision and recall for *Compacta* with a yeast assembly and generated sets of simulated assemblies for Arabidopsis, mouse and yeast with plots of precision × recall for all comparisons (Section 2 in Additional file 1). We also compared differential expression patterns from various assemblies (Section 4 in Additional file 1). Together these results showed that Compacta efficiently detected differential expression patterns after assembly clustering.

## Conclusions

In most cases de novo assemblies produce an excess number of contigs, many of which represent minor transcription variants from expression of the same gene. We assert that without full genome information segregating contigs by gene of origin is very difficult, if not impossible. Thus, for genomes that do not have complete information, researchers must reduce the analytical complexity by selecting a set of contigs to represent the entire transcriptome. *Compacta* provides flexibility in the selection of sets of representative contigs for downstream analysis. Its dynamic range goes from maximum transcriptome compression –wherein all contigs that share common reads are represented by a single contig, down to minimum compression, wherein only those contigs that are subsets of the same reads are clustered together and thus represented by a single contig. Because *Compacta* is fast, many test runs can be made to find the optimum level of transcriptome compactness according to the specific aim of a given experiment.

## Availability and requirements

**Project name:** Compacta.

**Project home Page:** https://github.com/bioCompU/Compacta or 10.5281/zenodo.3469484 Home page includes a source code tar ball, compiled executables and a software manual with demos.

**Operating Systems:** any Unix-based system with proper installation, or Linux × 86 with standalone executable.

**Programming Language:** C++.

**Other Requirements:** Samtools, zlib library and a C++ compiler.

**Recommended Hardware:** The memory needed to run the software is determined by the size of the largest input BAM file. *Compacta* loads the input files in memory and releases them after data pre-processing. As such, when the input data are loaded, only a small amount of memory is needed for processing. For a given dataset *Compacta* typically requires more memory than that of tools such as *CD-HIT-EST*, but less than that needed by *Corset* and transcriptome assemblers. The experiments shown in this study were performed on a computer with 64 Gb RAM, and an Intel CoreTM™ processor i7–6700 with 4 cores.

**License:** GNU GPL version 3.

**Restrictions for use by non-academics:** None.

## Supplementary information

**Additional file 1.** Supplementary results. Additional text and figures are ordered according to sections in the main text.

## Data Availability

The datasets used in this study are available in the Sequence Read Archive (SRA) with accession numbers SRP043494, ERP016911 and SRP149554 for Mango, Arabidopsis and Mouse datasets, respectively. The source code and standalone executable of the version of Compacta used in this study are available at 10.5281/zenodo.3469484.

## Abbreviations

BAM: Binary Alignment Map
bp: base pair
cDNA: complementary DNA
*Ef*: Efficiency
mRNA: messenger RNA
