# Supplementary material for: Compacta: a fast contig clustering tool for de novo assembled transcriptomes
Source: BMC Genomics. 2020 Feb 11;21:148. doi: 10.1186/s12864-020-6528-x (PMC7014741; doi:10.1186/s12864-020-6528-x)
Supplement: Supplementary file 1 — Additional file 1. Supplementary results. Additional text and figures are ordered according to sections in the main text. [file 12864_2020_6528_MOESM1_ESM.pdf]

SUPPLEMENTARY RESULTS FOR
'Compacta: A fast contig clustering tool for de novo assembled transcriptomes'

FERNANDO G. RAZO-MENDIVIL [(1)], OCTAVIO MARTÍNEZ [(2)], AND CORINA HAYANO-KANASHIRO [(1)]

(1) - Departamento de Investigaciones Científicas y Tecnológicas de la Universidad de Sonora, Universidad de Sonora, Hermosillo, México.

(2) - Unidad de Genómica Avanzada (Langebio), Centro de Investigación y de Estudios Avanzados del Instituto Politécnico Nacional (Cinvestav), Irapuato, México.

Contents

1. A simple example of *Compacta*'s algorithm 1
2. Comparing *Compacta* with other clustering algorithms 3
3. Compacta performance with a *de novo* assembled transcriptome 9
4. Comparing differential expression 13
References 15

1. A simple example of *Compacta*'s algorithm

Here we present an example of the *Compacta* algorithm for a simple set of simulated data. Figure 1 graphically summarizes the information present in the BAM file for three contigs, c_1, c_2 and c_3 (black lines in the first row of the figure), and the reads that the assembler used to construct such contigs (colored lines).

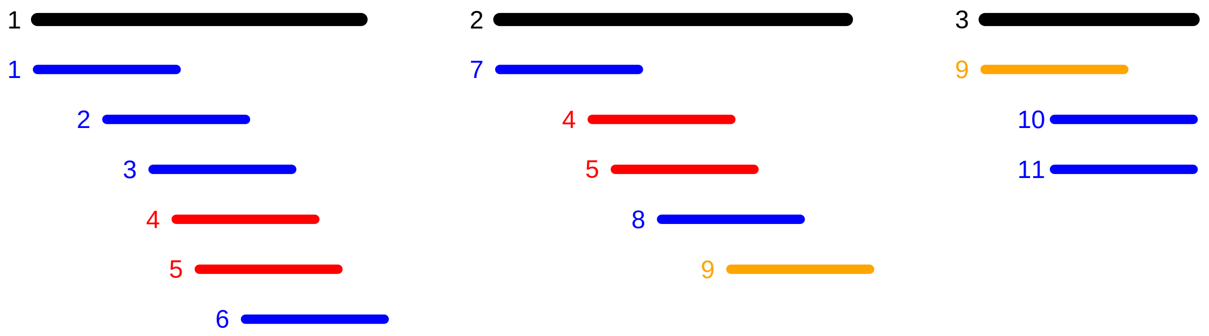

Figure 1. **Contigs to be cluster by *Compacta*.** Black lines represent contigs c_1, c_2 and c_3. Colored lines represent reads r_1 to r_{11}, and the plot shows how each read maps to the contigs. Blue lines are reads unique to each contig, red lines are reads shared by contigs c_1 and c_2 while orange line is read shared by contigs c_2 and c_3.

Before proceeding, it is worth noting that the information present in the BAM file, and graphically summarized in Figure 1, will be reduced by ignoring the exact position of each read at each contig, considering contigs only as subsets of all the reads, which are the 'universal set' of the problem, say $\Omega = \{r_1, r_2, \cdots, r_1 1\}$. Thus, *Compacta* will consider contigs as subsets of reads, say $c_1 = \{r_1, r_2, r_3, r_4, r_5, r_6\}$, $c_2 = \{r_4, r_5, r_7, r_8, r_9\}$ and $c_3 = \{r_9, r_{10}, r_{11}\}$, while the information about contig and read lengths will be stored separately.

Date: January 6, 2020.

We modeled using unpaired reads of 150 bp, and Figure 1 shows how each read maps to the contigs. Blue lines (reads $r_1, r_2, r_3, r_6, r_7, r_8, r_{10}$ and r_{11}) are reads that map to a single contig, r_1, r_2, r_3 and r_6 to contig c_1, reads r_7 and r_8 to contig c_2 and reads r_{10} and r_{11} to contig c_3. On the other hand, reads r_4 and r_5 (red lines) map to both contigs c_1 and c_2, while read r_9 (orange line) is shared by contigs c_2 and c_3, i.e., this read maps to both contigs. Table 1 presents the characteristics of the contigs that *Compacta* take into account for the step '**Filtering of low evidence contigs**' of the algorithm.

Table 1. Length, number of reads and effective coverage for the contigs presented in Figure 1.

Contig	Length (bp)	Number of reads	Effective coverage
c_1	375	6	$(6 \times 150)/375 = 2.400$
c_2	400	5	$(5 \times 150)/400 = 1.875$
c_3	225	3	$(3 \times 150)/225 = 2.000$

In contrast with other programs for contig clustering, as *Corset* (Davidson and Oshlack, 2014) or *Grouper* (Malik et al., 2018), *Compacta* do not simply set a uniform threshold on the number of reads per contig for them to be clustered, but takes into account the *effective contig coverage* which is the sum of the lengths of all reads group in the contig divided by its length. Given that we used reads of 150 bp, we can see that the effective coverage of contigs c_1, c_2 and c_3 is 2.4, 1.875 and 2, respectively.

If this example is run in compacta with the default parameter "`-l 2`", i.e., asking for a minimum effective coverage of $l = 2$, then contig c_2 will not surpass this threshold ($1.875 < 2$) and it will be marked as a 'low evidence contig' and output as a singleton, without entering into the *Compacta* clustering algorithm. In what follows we assume that compacta was run with parameters "`-l 1.5 -d 0.3`", and thus the three contigs in the example will pass the minimum effective coverage filter and enter into the pre-cluster (step number 3) of the *Compacta* algorithm.

In *Compacta* the formula to compute the weight w_{ij} between contigs i and j is given by

$$w_{ij} = \frac{R_{ij}}{\min(R_i, R_j)}$$

where R_i and R_j are the number of reads mapping to contigs i and j, respectively, and R_{ij} is the total number of reads that map to both contigs i and j. Table 2 shows the calculation of the weights, w_{ij}, between the set of three contigs previously shown in Figure 1.

Table 2. Calculation of weights, w_{ij}, for all pairs of contigs in the graph calculation.

Contig i	Contig j	w_{ij}
c_1	c_2	$2/\min(6,5) = 2/5 = 0.40$
c_2	c_3	$1/\min(6,3) = 1/3 = 0.33$
c_1	c_3	0 given that $R_{1,3} = 0$

Tp proceed with the treatment that *Compacta* will give to the input data it is important to note that contigs c_1, c_2, c_3 form a closed subgraph, because we have reads that connect the three contigs, say $c_1 \leftrightarrow c_2 \leftrightarrow c_3$ (even when c_1 is not directly connected with c_3). Thus, in step 4 of the algorithm (**Pre-cluster detection**), *Compacta* will detect the fact that the contig set $\{c_1,\ c_2,\ c_3\}$ forms a pre-cluster, and will load the information about the weight of all the contig pairs into an auto sorted heap structure, in the decreasing weight order which it is presented in Table 2.

In the **Clustering** step, the largest weight between contigs, that in this case corresponds to the weight between c_1 and c_2, say $w_{1,2} = 0.40$, will be compared with the threshold input by the user, `-d 0.3`; $d = 0.3$ Given that we have that $w_{1,2} = 0.40 > 0.3$, contigs c_1, c_2 will form the first cluster, say $C_1 = c_1 \cup c_2$. We have that

$$C_1 = c_1 \cup c_2 = \{r_1, r_2, r_3, r_4, r_5, r_6\} \cup \{r_4, r_5, r_7, r_8, r_9\} = \{r_1, r_2, r_3, r_4, r_5, r_6, r_7, r_8, r_9\}$$

thus now the cluster C_1 contains 9 reads that previously were in c_1 *or* c_2.

The clustering step proceeds by calculating the weights between the elements currently present in the pre-cluster, say the new cluster $C_1 = c_1 \cup c_2$ and the original contig c_3. That weight is $w_{C1,c3} = 1/\min(9,3) = 1/3 = 0.33$. Given that this weight is larger than the threshold $d = 0.3$, a new cluster, say $C_2 = C_1 \cup c_3 = \{r_1, r_2, \cdots, r_{11}\}$ is formed, and this new cluster includes all reads present in the example and will be reported in the output. The following text shows the structure of the output files for this example when run with parameters `-d 0.3 -l 1.5`

```
--------------------------------------------------------------------------------
## Cluster definition file:
contig.1     Cluster.1
contig.2     Cluster.1
contig.3     Cluster.1
--------------------------------------------------------------------------------
```

where `contig.1` $= c_1$, `contig.2` $= c_2$ and `contig.3` $= c_3$ and the final result shown above, say C_3 is re-named here as `Cluster.1`

```
--------------------------------------------------------------------------------
## Raw counts per cluster file:
Cluster.1     11
--------------------------------------------------------------------------------
```

In contrast, if the example is run with the default parameters, `-d 0.3 -l 2`, then the *Compacta* algorithm will not group any of the three original contigs into clusters, because c_2 will not pass the threshold $l = 2$, and thus pre-cluster $\{c_1, c_2, c_3\}$ will not exist and c_2 will be reported as a 'singleton', while c_1 and c_3 will be reported as 'clusters with a single contig'.

2. Comparing *Compacta* with other clustering algorithms

In this section we include additional tests performed to evaluate the *Compacta*, *Corset* and *Grouper* algorithms using sub-sets of data, created from the Arabidopsis and mouse transcriptomes (Table 3), using the new tuxedo pipeline (Pertea et al., 2016). First we aligned the reads to their respective genome using *Hisat2* (Kim et al., 2015) with default parameters. The '`--dta`' option was used to allow compatibility with the *Stringtie* assembler, and the '`--known-splicesite-infile`' option was used with the splicing sites extracted from the annotation file. The output files were sorted and converted to `bam` format using *Samtools* 1.5 (Li et al., 2009). Then we used *StringTie* (Pertea et al., 2015) to estimate transcripts abundances on the `bam` alignment files using parameter '`-G`' to include the annotation file, '`-B`' to output tables compatible with *Ballgown* and '*-e*' to limit the read estimation only to transcripts matching the reference. We used two *Bioconductor* (Huber et al., 2015) packages, *Ballgown* (Frazee et al., 2014) to load the abundance matrix created with *Stringtie*, and *Polyester* (Frazee et al., 2015) to simulate two treatments with 30 million paired-end reads for each organism based on the abundances of the real datasets obtained with *Stringtie* and *Ballgown.* Only samples ERR1592576 and ERR1592579, SRR7248743 and SRR7248745 of the Arabidopsis and mouse datasets,respectively,were used to create the simulated datasets. Additionally, we created two extra datasets based on the abundances obtained from the real data of Arabidopsis to test the effect of coverage on the clustering efficiency, each set includes two treatments with 10 and 50 million paired end reads per sample,respectively.

Polyester output consists in fasta files without quality scores or sequencing adapters to remove so we skipped the Quality Control step and proceeded to the *de novo* assembly using Trinity with default parameters including the '`--seqType fa`' flag to specify the input format, the number of contigs produced by *Trinity* for each organism can be seen in Table 3. Simulated reads were mapped back to their respective transcriptome as paired-end using *Hisat 2* with parameters '`-f -k 100 --score-min L,-0.1,-0.1 --no-spliced-alignment`', obtaining at least an 88% mapping rate per sample. We used

Salmon v0.12.0 (Patro et al., 2017) with options '`--dumpEq --writeOrphanLinks`' to pseudo map clean reads against their respective transcriptome in order to obtain the equivalence classes needed to run Grouper, a mapping rate of at least 95% was obtained for each sample.

TABLE 3. Simulated datasets of Arabidopsis and Mouse produced by Polyester, and contigs in the *de novo* assembled transcriptome produced by Trinity.

Organism	Number of samples	Number of reads	Assembled contigs
Arabidopsis	2, paired-end.	27,933,799	35,785
		27,620,029	
Mouse	2, paired-end	18,701,459	32,212
		19,072,074	

Output files created by *Hisat2* and *bowtie2* were converted to BAM format (Li et al., 2009) using *Samtools* 1.5 (Li et al., 2009), BAM files were processed with corset 1.07 using default parameters adjusting group definition (*-g* option) according to samples in each experiment, and we set parameter '`-D 9999999999`' to disable paralog detection. BAM files were then processed with *Compacta* 1.0 using default parameters, adjusting '`-n`', '`-g`' and '`-s`' options to match the structure of each dataset. We clustered the *de novo* assembled transcriptomes using *CD-HIT* v4.7 (Li and Godzik, 2006) with parameters '`-c 0.95 -n 8 -T 0 -g 1 -d 0`'. To obtain the assembler cluster list we used the script `get_Trinity_gene_to_trans_map.pl` from *Trinity* on each of the assembled transcriptomes to create a list of contigs per cluster. Finally, clustering with *Grouper* was carried out using the equivalence class files produced by *Salmon* as input and default parameters with '`orphan: True mincut: True`'.

Figure 2 shows the execution time in seconds for Compacta, Corset and Grouper on each of the simulated datasets, Trinity and CD-HIT were not included as their running time is not fairly comparable with the other clustering tools. In this figure each bar represents the running time of each program on one of the assemblies, x-axis groups bars by program and colors identify the assembly of each organism, Compacta is faster clustering both the Arabidopsis and the mouse simulated dataset than Corset -even thought the difference is not as remarkable as with the real datasets, comparing Compacta *vs* Grouper, we can see that Grouper is faster on the Arabidopsis simulated dataset, this can be explained by the fact that experiments with low-complexity are easy to cluster for the three algorithms, however, both Compacta and Corset load BAM files which consists in Gigabytes of information that must be parsed before clustering, while Grouper loads Equivalence files, smaller plain text files that are easily and quickly parsed; still, on the mouse simulated dataset Compacta was the fastest while Grouper was the slowest of the three programs, just as with the real datasets this shows that on tests with more complex data the time required to load and parse the input files becomes insignificant compared to the time required by the clustering algorithm, proving that Compacta is more efficient and makes a better use of the computer resources compared to Corset and Grouper.

Just like with the real datasets (see Figure 1 in the main text), we calculated recall (R), and precision (P) for the 5 clustering algorithms, 3 of which are 'read-based': *Compacta*, *Corset* and *Grouper*, plus *CD-HIT*, which is based on information from the contigs sequences, and *Trinity*, which is based on De Bruin graphs. All five algorithms were tested using the assemblies of the simulated datasets of Arabidopsis and mouse described previously, the five programs were run using default parameters and the results are shown in Figure 3.

In Figure 3 we can see that as with the real datasets, *CD-HIT* was the program with the poorest recall and the highest precision, again, this can be explained by the fact that *CD-HIT* uses information from the contigs sequences, this allows it to group highly similar contigs which rises the precision, it is worth to notice that precision is only calculated on the clustered contigs even though *CD-HIT* produces the smaller number of clusters it has a high precision; this approach to clustering leads *CD-HIT* to fail to group contigs related that vary at the sequence level, in fact, *CD-HIT* has trouble identifying alleles, and genes with SNPs and INDELs and it might produce different clusters for each one even thought

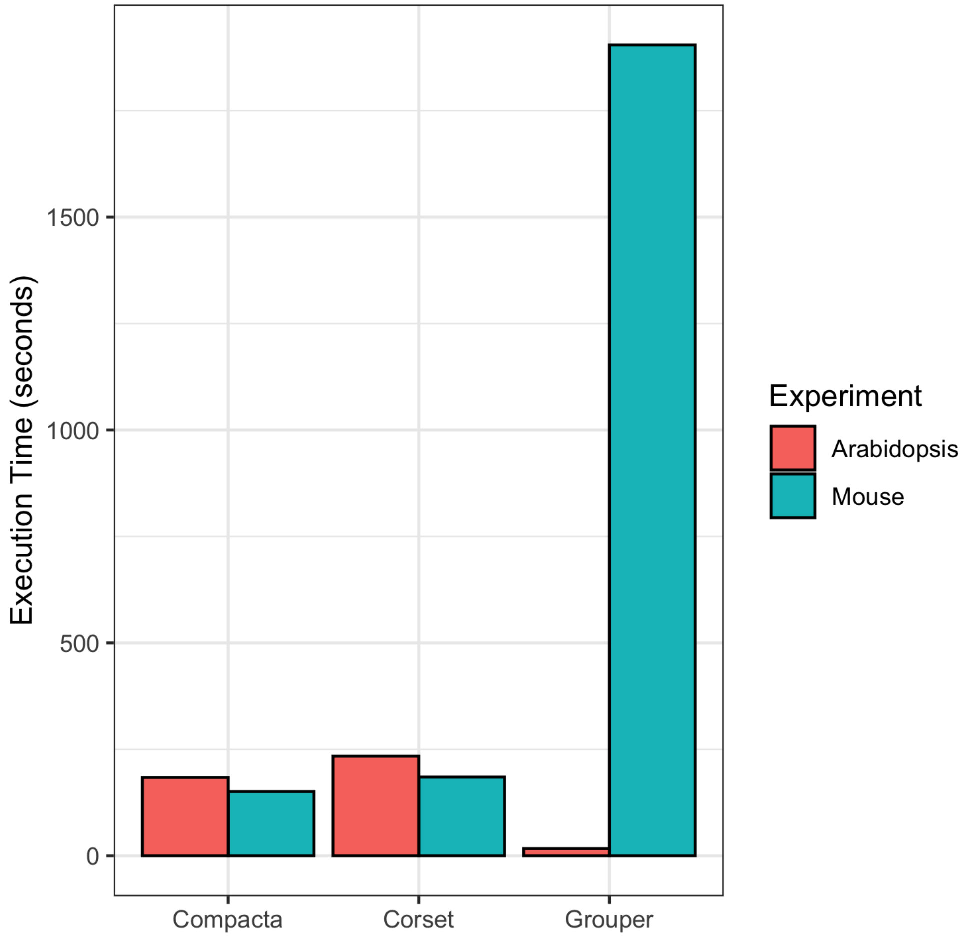

Figure 2. **Execution time evaluation.** Running time in seconds of Compacta, Corset and Grouper on simulated datasets of Arabidopsis and Mouse.

they should be together, this explains the low recall. However, read-based algorithms such as *Compacta*, *Corset* and *Grouper* seem to be more robust against the problems found in *CD-HIT*, in fact, the three algorithms show a more balanced precision and recall, in both test *Corset* posses a higher precision of the three programs, but it also shows the lowest recall of the three; *Grouper* competes with *Compacta* for the highest recall of the five algorithms, nevertheless, *Grouper* has an unexplained drop of precision in the mouse test; on the other hand, *Compacta* has shown greatest recall than almost all algorithms and a precision that is slightly lower than programs like *CD-HIT* or *Corset* but in general we think it has a good balance of precision and recall.

We created data sets to evaluate the behavior of the three read-based algorithms: *Compacta*, *Corset* and *Grouper* under different sequencing deep. With this aim we used three sets of simulated data of Arabidopsis at different sequencing levels, say, 10, 30 and 50 million reads, respectively. After mapping the reads back to their respective transcriptome, clustering with the three algorithms was performed and precision and recall were estimated. Figure 4 presents the results of these tests.

In Figure 4 we can see that the three programs show a stable precision through the three assemblies with different sequencing level, however, both, *Grouper* and *Corset*, show differences in recall across the three experiments, with an apparent decrease of recall as the sequencing level increases. In contrast, *Compacta* not only presents a stable precision on the three experiments, but it also shows a slightly increase of recall as the sequencing level increases. This implies that *Compacta* is not only faster than *Corset* and

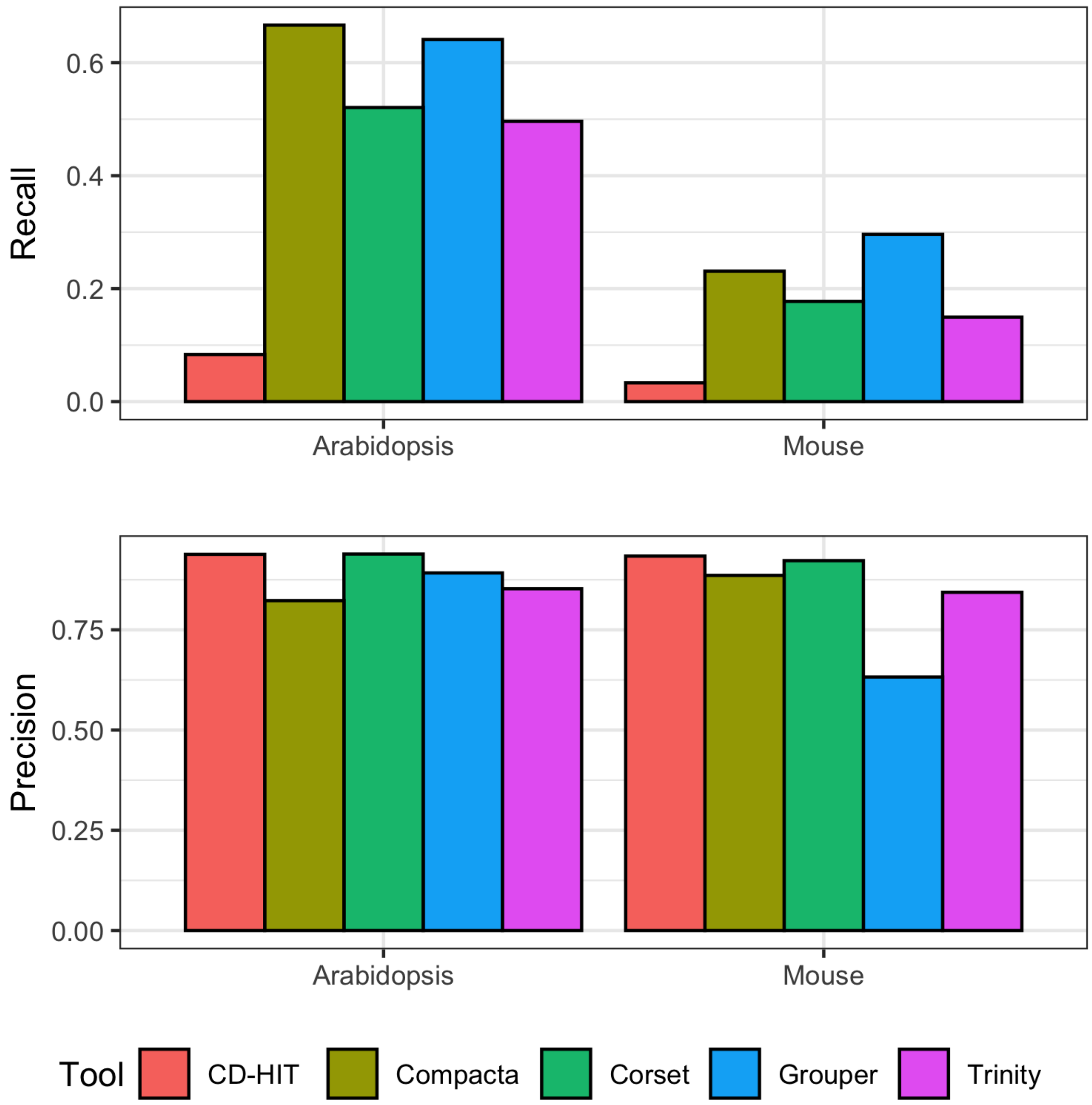

FIGURE 3. **Performance of different clustering software.** Precision and recall metrics estimated on clustering output of five clustering tools: *Compacta*, *Corset*, *Grouper*, *Trinity assembler* and *CD-HIT* on Arabidopsis and mouse realistically simulated datasets.

Grouper on complex datasets, giving a well balanced precision-recall, but also it might have a more stable behavior (precision and recall) as the number of reads increases. These results show that *Compacta* is a more robust algorithm than the ones implemented in *Corset* and *Grouper*.

We have previously shown how the parameter d can affect the number of clusters and the number of contigs per cluster produced by *Compacta*, but this is not the only characteristics affected by this parameter as changing the cluster configurations might affect the performance of the clustering algorithm. In order to evaluate the effect of the cut off value (parameter d), in the performance of *Compacta*, we used the simulated dataset of Arabidopsis with 30 million reads, and ran *Compacta* with default parameters using values of d from 0.1 to 1 with increments of 0.1, then we calculated precision and recall from the outputs produced by the algorithm. Results of these tests are shown in Figure 5.

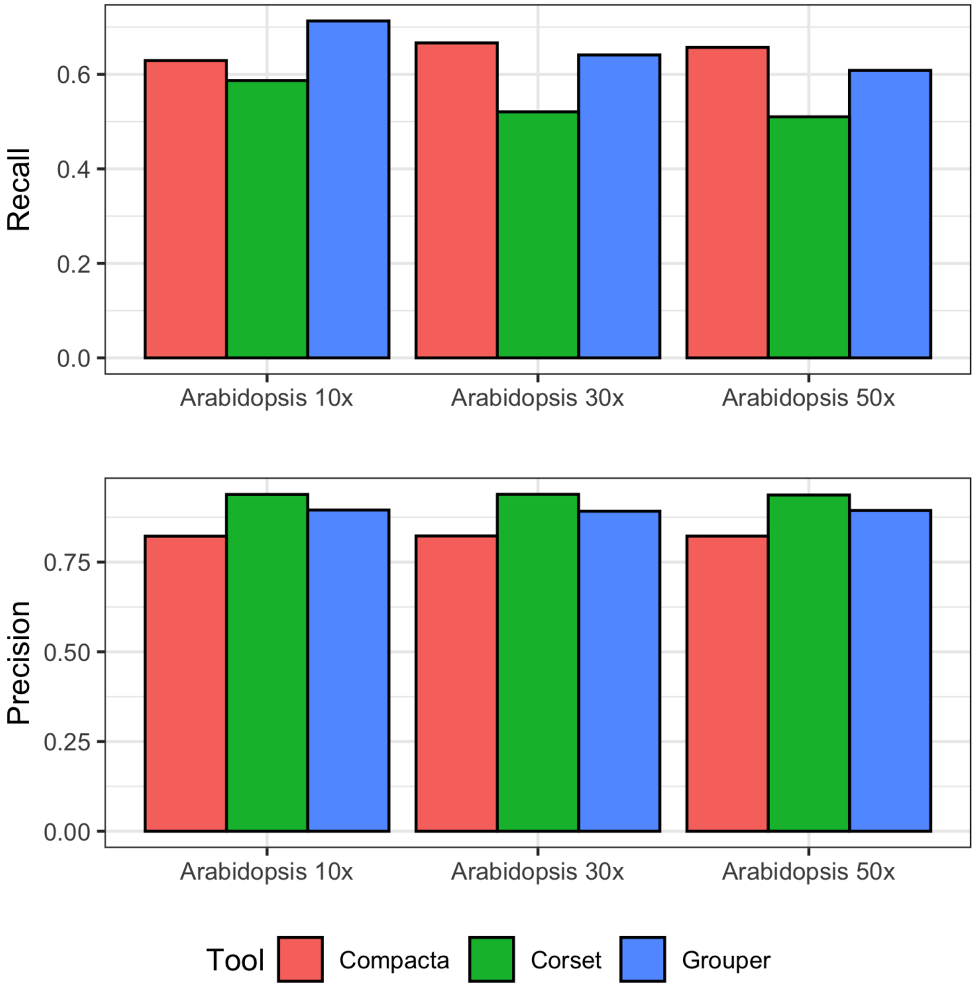

FIGURE 4. **Impact of sequencing deep on the clustering performance.** Three experiments of Arabidopsis realistic simulated datasets with 10, 30 and 50 Million pair-end reads per sample, clustered using *Compacta*, *Corset* and *Grouper* shows that sequencing deep has an impact on the clustering performance. *Compacta* recall increases with the number of reads and manages to maintain stable after 30 Million reads, while *Corsets* recall decreases as the number of reads per sample increases.

As we can see in Figure 5, the first test (0.1) posses the highest recall and the lowest precision, and as we increase the value of d the precision increases but some recall is lost in process, finally, with a d of 1 both precision and specially recall drops, because the threshold is so small that only contigs sharing lots of reads are clustered together, and some of the related contigs are being left out without being clustered, we choose to use a d value that produces a balanced values of recall and precision, in this case we used 0.3 as the value of d, but other values like 0.2 or 0.4 would be as good, depending on the user preferences.

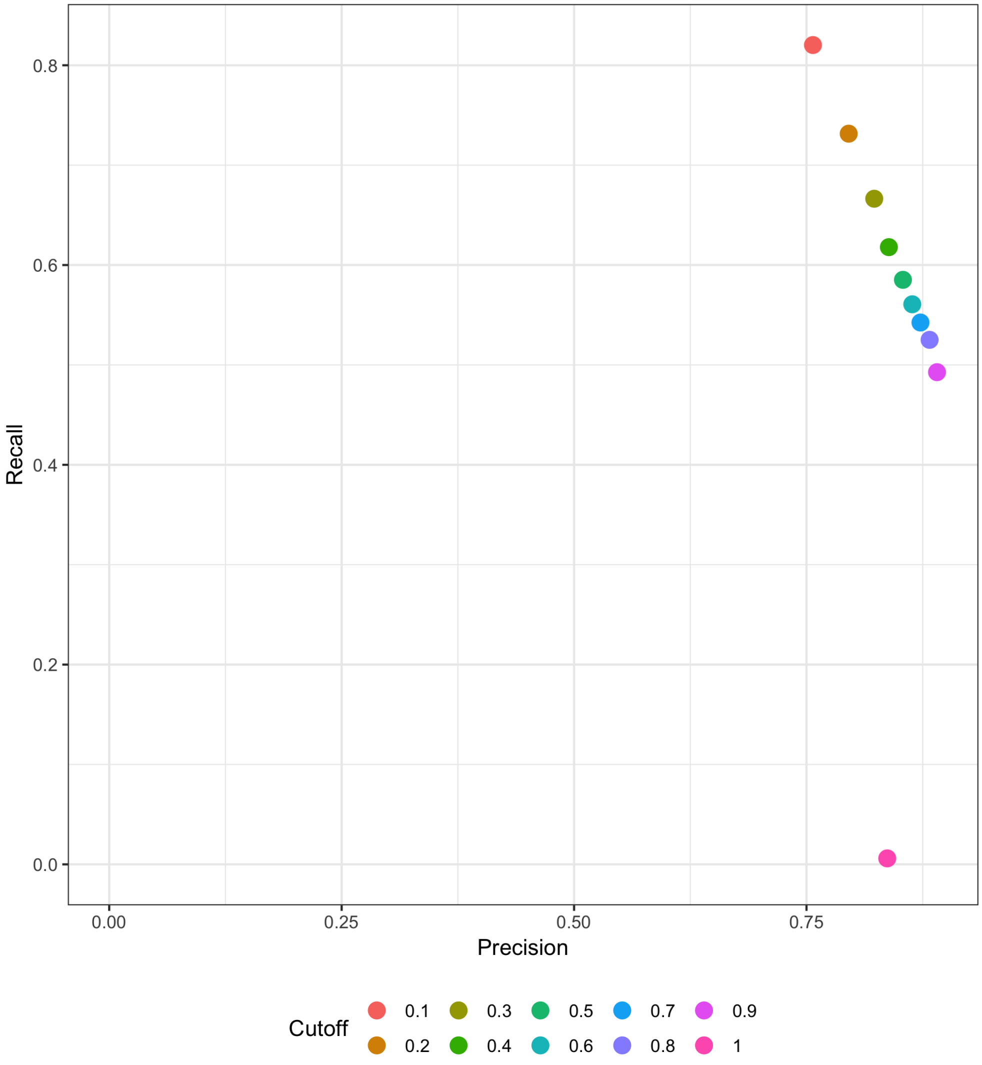

FIGURE 5. **Effect of parameter d on precision and recall.** Precision and Recall of ten runs of *Compacta* on the Arabidopsis simulated dataset using different clustering threshold values (parameter d), when we choose a threshold value closer to zero the algorithm clusters more contigs rising up the Recall but loosing precision, and when we choose a threshold value closer to one the algorithm gets more strict on choosing which contis to cluster which reflects in a greater precision but leaving out many contigs dropping the recall.

3. Compacta performance with a *de novo* assembled transcriptome

The Arabidopsis transcriptome employed here came from the experiment in (Liu et al., 2016). The full experiment produced approximately 36 GB of high-quality reads. For the proposes of *Compacta* evaluation these reads were assembled *de novo* using Trinity v2.4.0 (G Grabherr et al., 2011) with default parameters. The assembly gave a total of 106,895 contigs with length varying from 201 to 18663 bp with mean and median sizes of 1987 and 1636, respectively. The sum of the length of all contigs was ≈ 212.4084 Mb, while the standard deviation of contig size was $\hat{S} \approx 1626$.

The figures above give evidence of the problem that arises with *de novo* transcriptome. We have 106,895 contigs; however, the true maximum of sequences that can be produced by the Arabidopsis genome is 41,671, thus the 'redundancy' in the assembled transcriptome is at least, $106895/41671 \approx 2.6$, and that is assuming that absolutely all 33,602 Arabidopsis genes were expressed in the experiment in all the 41,671 possible variant forms. Of course, that assumption is highly unlikely, thus we can be sure that the redundancy of sequences in the assembly must be > 2.6

In this section we will consider the number of clusters given by *Compacta*, say z, as a function only of the parameter `-d`, say $z = f(d)$. The parameter d, which can vary from zero to one, gives a threshold to consider that two contigs (in the input) belong to the same cluster (in output), and it controls clustering extent. Thus, with a value of $d = 0$, all contigs that share one or more reads will be cluster, while with $d = 1$ only contigs that are subsets one of the other will be reported as clusters.

Compacta was applied to the pre-processed files ('pre-clusters') of the transcriptome, which in turn were obtained from the original BAM files downloaded from the source of the transcriptome, see (Liu et al., 2016). *Compacta* was run with a grid for the parameter `-d` from 0 to 1 in 0.1 increments (11 values). Then, a finer grid was tried near the points where the slope of the function $z = f(d)$ had a sudden change. Table 4 gives values of z for selected values of d, while Figure 1 in the main text shows the estimated function $z = f(d)$ (as percentage) for the transcriptome analyzed.

Table 4. Number of clusters, z, given by *Compacta* for selected values of d.

d	z	$\approx$ Slope $z = f(d)$	$\%max$	$z/\min(z)$
0.000	13770	≈ 934600	13.34	1.00
0.035	28704	90800	27.80	2.08
0.500	34860	4600	33.76	2.53
0.955	40656	89800	39.37	2.95
1.000	103262	8137600	100.00	7.50

In Table 4 we can see that the value of z can vary from $z_{min} = 13770$ when $d = 0$, to $z_{max} = 103262$ when $d = 1$. Given that the number of contigs input to the algorithm was 106895, a total of $106895 - 103262 = 3633$ of the original contigs where either, filtered by presenting a low coverage (the value of parameter `-l` was set to the default value of 2), or considered as fully redundant contigs. The interval $(13770, 103262)$ gives the range of values of z that can be approximated by varying the value of d. If we consider a linear function $z = a + bd$ –which is shown as a dashed grey line in Figure 2 of the main text, that straight line will have values of intercept $a = 13770$ and slope $b = (103262 - 13770)/1 = 89492$. However, as shown in Figure 2 of the main text, the function $z = f(d)$ is very different from an straight line. For values in $0 \leq d \leq 0.03$ the $f(d)$ curve has a very steep slope, which suffer a sudden change at approximately $d = 0.35$. For values in approximately $0.035 \leq d \leq 0.9$, the slope of $f(d)$ is relatively small, i.e., the number of clusters z given by the algorithm increases slowly as function of d. This behavior changes suddenly around $d \approx 0.955$, and from there on ($d > 0.956$), the function has again a very steep slope. In Table 4 we can see the approximate estimated slope of the $z = f(d)$ curve at the d points (column '$\approx$ Slope $z = f(d)$'). Near the 'critical' points $d = 0.035$ and $d = 0.955$ is where the estimated slope of the curve (90800 and 89800, respectively are closer to the slope assuming linear behavior, $b = 89492$. Column $\%max$ in Table 4 shows the percentage of z with reference to the maximum number of clusters that can

be obtained with *Compacta*, i.e., the percentage of z with reference to103262 (obtained with $d = 1$). At $d = 0$ the value of $\%max$ is 13.34, indicating that there is not possible to select a smaller percentage of contigs to represent the full collection. On the other hand, the last column in Table 4, $z/\min(z)$, shows the level of 'compression' achieved by the algorithm with different values of d, for example, with $d = 1$ we have 7.5 more clusters than the minimum number obtained $d = 0$, etc.

Even when in Figure 2 of the main text the function $z = f(d)$ resembles a continuous function, it is obvious that it is in fact a non-decreasing step function of the continuous threshold d. It is a step function because the value of z –the number of clusters, is a discrete quantity, while it is non-decreasing because if at d_1 we have that $f(d_1) = z_1$, then for every $d_1 + \epsilon$ with $\epsilon > 0$ and $d_1 + \epsilon \leq 1$, it follows that $f(d_1 + \epsilon) \geq z_1$, i.e., if two or more contigs had been clustered with a value of d_1, they will remain clustered at $d_1 + \epsilon$ and thus the total number of clusters, z, must be the same or increase, but it will never decrease (when two contigs had been integrated into a cluster, there is no way in which they could be segregated by a further step of the algorithm).

The exact form of the function $z = f(d)$ is 'transcriptome dependent' –meaning that it is the result of various factors, including genome structure, 'treatments' under which the transcriptome was obtained (tissues, conditions, etc.), the gene expression profile under each 'treatment' as well as the length and number of reads employed to obtain the transcriptome. Let's assume that the sequencing depth of each one of the libraries is not limiting; i.e., that even the genes with lower expression are represented in the transcriptome by at least one contig. Then, the size of the reads employed will be the most important factor to confound two of more contigs that are generated from two or more different loci. For example, if the the transcriptome is fully sequenced with very log reads, as the ones generated by the PacBio technology (Rhoads and Au, 2015), we could have that all different transcripts will be shorter than the average read length and then there will be not 'shared' reads between contigs and thus no need to apply any algorithm for contig clustering; in fact, in that case the function $z = f(d)$ will be just a constant z^*, independent of the values of d. With reads smaller than the average size of the transcripts it becomes possible that some reads will be shared between two or more of the assembled contigs; the corresponding contigs will be linked by reads representing motifs shared between alleles, splicing variants, post-transcriptional modifications, etc. Having all other conditions constant, smaller reads will cause a larger number of contigs in the output of the assembler and in that case algorithms such as *Compacta* will be useful to select a representative set of contigs (clusters) for downstream analyses.

The relevant question is how to select the value of d –and of course, it depends to some extent of the aim of the research project. If the researcher wants to perform a very general analysis, for example, to identify the main genes or groups of genes represented in the transcriptome, then a value of $d = 0$ will give the maximum transcriptome 'compression' by selecting only one contig from each group. On the other hand, if a very detailed analysis is desired, a value of $d = 1$ will filter only contigs with low coverage selecting all different transcripts, except those that are subsets of each other.

A third and more frequent case is that the researcher wants a set of contigs that represents the most relevant groups of transcripts with a relatively low level of redundancy. In such case the researcher needs some guidance about the value of d that will give such set. An advantage of using the Arabidopsis transcriptome is that its genome is very well annotated; of course, that benefit will be absent in all cases where the transcriptome came from an unknown (not sequenced) genome. However, investigating the number of loci expressed in the example transcriptome will suggest general guidelines for the selection of a suitable value of d in the general case.

3.0.1. *The set to compare the Arabidopsis transcriptome.* To compare the assembly as well as *Compacta* results we downloaded from TAIR10 blastsets the file `TAIR10_cdna_20101214_updated`, which contains all cDNA's known from Arabidopsis (defined as CDS+UTRs-introns). The Arabidopsis cDNA file contains 41,671 sequences, corresponding to 33,602 loci, thus, in average, each locus is represented by $41671/33602 \approx 1.24$ sequences. This cDNA sequences were formatted as a BLAST DB named here 'TAIR10cdna'. Note that in the guiedelines for Arabidopsis nomenclature, each locus is identified by

a unique chain of symbols, for example as '`AT1G51370.2`', meaning '`AT`' = *Arabidopsis thaliana*, '`1`' = chromosome 1, '`G51370`' = gene 51370 and, finally '`.2`' represents the second sequence from that locus (`G51370`).

The 106895 contigs in the Arabidopsis assembled transcriptome were used as queries to the BLAST database TAIR10cdna of all 41671 sequences of cDNA's from this plant. The BLAST experiment was performed with an *in situ* installation of the NCBI BLAST implementation [Package: blast 2.7.1, build Oct 18 2017] (Zhang et al., 2000). The command line for the blast experiment was

```
blastn -query allContigs.fasta -db TAIR10cdna -out allContigs_out1.txt -outfmt 6
       -dust no -max_target_seqs 1 -ungapped
```

This command line implies the use of the task 'megablast' in tabular format with no 'dust' filter and giving only one result for each one of the contigs queried. Only continuous (non-gapped) hits were taken into account. The resulting file '`allContigs_out1.txt`' was included into the table '`allContigs_out1`' of the MySQL DB '`razo`'.

The results in '`allContigs_out1`' included 194,641 rows for 94,286 contigs having hits to 27,016 distinct Arabidopsis sequences. A total of $106895 - 94286 = 12609$ contigs ($\approx 12\%$ of the total) did not gave any hit, showing that such contigs could be, either, assembly artifacts or transcripts from post-transcriptional modifications which are not currently annotated in Arabidopsis.

Next step in the analysis was to select only the hit with maximum bit score, i.e., the most 'significant' for each pair of contig / Arabidopsis sequence. These results are in table '`contigs2locus`', and consist in 94,286 pairs (concordances contig / Arabidopsis sequence) for 27016 different Arabidopsis sequences. This implies that we have in average $94286/27016 \approx 3.49$ contigs for each one of the Arabidopsis sequences.

The next problem was to determine which of these 94286 pairs (concordances contig / Arabidopsis sequence) where relevant or 'significant' in the sense that a given contig was appropriately representing an Arabidopsis locus. To consider a BLAST result as relevant we asked that the length of the alignment between the contig and the Arabidopsis sequence include a minimum of 75% of the contig OR a minimum of 75% of the Arabidopsis sequence AND presents a minimum bit score of 90. This last requirement implies that the maximum expected value (BLAST 'E-value') was 1.47×10^{-16}, i.e., extremely significant from the statistical point of view. Only 48311 of the 94286 pairs ($\approx 51\%$) of such concordances fulfilled the criteria (results in table '`relevant_relations`' in the DB).

From the results in table '`relevant_relations`' we conclude that 48311 contigs represent 23607 Arabidopsis sequences, that in turn came from 20934 different loci. The difference between 23607 and 20934 arises because each locus can produce various transcripts; in this case we have that in average each locus is represented by $23607/20934 \approx 1.13$ Arabidopsis sequences.

From these BLAST experiment we can conclude that in principle a very small number of contigs, ≈ 23607, could be sufficient to represent the same number of different Arabidopsis sequences expressed within the transcriptome. That target number of contigs is shown as a violet arrow in Figure 1, and it implies that a very small value of d, say $d \approx 0.01$, could give such small number of representative contigs. However, in principle nothing guarantee that the z contigs selected by *Compacta* will reasonably represent all 23607 Arabidopsis sequences. To study that problem it is necessary to see how many Arabidopsis sequences or loci are represented by the z contigs selected with a value of d.

Table 5presents the selected number of contigs (n_{con}), number of Arabidopsis sequences represented (n_{As}) by having a bit score ≥ 90 (E-value $\leq 1.47 \times 10^{-16}$, the percentage of the total of Arabidopsis sequences represented ($\%As$) and the percentage of efficiency ($Ef = 100 \times n_{As}/n_{con}$) for different values of d. Also Figure 2 in the main text presents the plot of $\%As$ and $\%Ef$ as function of d.

From Table 5 we can see that both, n_{con} and n_{As} are increasing functions of d; larger values of d imply larger numbers of representative contigs selected and as consequence a larger number of clearly identified

TABLE 5. Selected number of contigs (n_{con}), Number of Arabidopsis sequences represented (n_{As}), Percentage of the total of Arabidopsis sequences represented ($\%As$) and Percentage of Efficiency ($Ef = 100 \times n_{As}/n_{con}$) for different values of d.

d	n_{con}	n_{As}	$\%As$	Ef
0.00	4361	3344	14.17	76.68
0.05	19962	18381	77.86	92.08
0.10	21102	19257	81.57	91.26
0.15	21674	19607	83.06	90.46
0.20	22085	19813	83.93	89.71
0.25	22451	19986	84.66	89.02
0.30	22778	20111	85.19	88.29
0.35	23061	20234	85.71	87.74
0.40	23256	20311	86.04	87.34
0.45	23489	20392	86.38	86.82
0.50	23670	20455	86.65	86.42
0.55	23919	20527	86.95	85.82
0.60	24120	20583	87.19	85.34
0.65	24349	20658	87.51	84.84
0.70	24577	20712	87.74	84.27
0.75	24847	20777	88.01	83.62
0.80	25181	20844	88.30	82.78
0.85	25632	20924	88.63	81.63
0.90	26351	21034	89.10	79.82
0.95	28207	21254	90.03	75.35
1.00	90477	23607	100.00	26.09

Arabidopsis sequences. Each contig employed is the largest one of the corresponding *Compacta*'s cluster. It is also important to note that, for all values of d, $n_{con} > n_{As}$; i.e., the number of contigs employed is always larger than the number of Arabidopsis sequences identified by them, which in turn means that there are some identification redundancy and not a one to one relation between contig and Arabidopsis sequence.

Also from Table 5, but more clearly in Figure 2 in the main text, we can see that $\%As$ is a not decreasing function of d –as $z = f(d)$ in Figure 2 in the main text. Also as $z = f(d)$ in Figure 2 in the main text, $\%As$ presents a very steep slope for $0 \geq d \leq 0.05$, going from 14.17 at $d = 0$ to 77.86% at $d = 0.05; \Delta\%As/\Delta d = 1273.8$ Thus at $d = 0.05$ we have 77.86% of all Arabidopsis sequences correctly identified. At $d = 0.05$ we have the maximum value of $Ef = 92.08$; at that point the number of identified sequences per contig peack, decreasing at relatively slow pace until $d = 0.75$, where it goes down fast to its minimum value of only 26.09 at $d = 1$.

From Figure 2 in the main text it is obvious that there is not a value of d which maximizes both, $\%As$ and Ef simultaneously. The selection of any d value means a compromise between number of contigs to be analyzed and the proportion of Arabidopsis sequences correctly identified in the transcriptome.

However, given that *Compacta* is fast and efficient, rounds of runs can be performed until a suitable value of d –and consequently a set of z contigs to be analyzed, can be heuristically determined to optimize the set which is best suited for the research objectives. Compared with the cost of data gathering (RNA-Seq library construction and sequencing), the cost of analyses is insignificant.

All results presented in this subsection are available from the authors as a relational database.

4. Comparing differential expression

Contig clustering may affect the gene expression analysis; poor precision caused by over-clustering can lead to problems detecting deferentially expressed genes because genes with different relative expression are clustered together, while poor recall caused by under-clustering increases the size of the reported transcriptome, making it difficult to execute downstream analysis Davidson and Oshlack (2014). To test the effect of *Compacta*'s clustering on gene expression analysis, we used the real datasets from Arabidopsis and Mouse, previously employed. We used *Hisat2* to map the reads back to their corresponding transcriptome and *Compacta* to cluster the contigs and produce cluster-contig lists and raw counts matrices. Then we used *RSEM* v1.3.1 on the cluster definitions produced by *Compacta* to build references with parameters '`rsem-prepare-reference --bowtie --transcript-to-gene-map`', and again, we used *RSEM* but this time to calculate the expression levels with parameters '`rsem-calculate-expression --estimate-rspd --paired-end`' to get cluster level counts. We also used *RSEM* on the same datasets but this time we build the references using the corresponding genome with parameters '`rsem-prepare-reference --bowtie --gtf`' then we calculated gene level expression with

'`rsem-calculate-expression --estimate-rspd --paired-end --no-qualities`'

to get a reference based on truth data.

We used the count matrices produced by *Compacta*, the cluster level counts and the gene level counts produced by *RSEM* in *EdgeR* to carry on differential gene expression analysis using the exact test with $FDR = 0.01$ and $\|lfc\| >= 1$ to detect significant differentially expressed genes. For each test we performed cluster identification against the corresponding genome using *Blast-n* v2.6.0 with parameters '`-max_target_seqs 1 -outfmt 6 -evalue 1e-3`' and discarded alignments with a length under 200 bp and below 98% identity. Also we ignored clusters without a match in the genome-based differentially expressed gene list and when there was more than one cluster per gene only the one with the biggest lfc value was picked. Then we used Pearson's and Spearman's correlation on the filtered significantly differentially expressed clusters obtained from *RSEM vs* the differentially expressed genes (genome-based) and on the DE clusters obtained from *Compacta* counts *vs* DE genes from the genome-based tests. Results are presented in Figure 6.

In Figure 6, subfigures A and B show the correlation on differentially expressed clusters of Arabidopsis using *RSEM* and *Compacta*, respectively, against the differentially expressed genes guided by the reference genome. Both graphs show a correlation greater than 0.96 with an $\alpha = 0.9$ The correlation values vary slightly in both tests. This means that statistically significant DE clusters obtained by using the count data output by *Compacta* in a Differentially Expressed gene analysis are very similar to those DE genes selected from a genome guided DE analysis, and these results are not different from using abundance estimating tools like *RSEM*. Subfigures C and D show the correlation of differentially expressed clusters of mouse using *RSEM* and *Compacta* as abundance estimating tools, respectively. We can see that they have a correlation against the differential gene expression analysis guided by the reference genome greater than 0.9 with an $\alpha = 0.9$. This test show correlation coefficients with smaller values than the ones in the Arabidopsis tests which can be explained by the complexity of the dataset. However, *Compacta* shows a slightly higher correlation coefficient (with a difference of 0.0103 and 0.0377 for Pearson's and Spearman's, respectively). The difference in *RSEM* correlation coefficient against *Compacta* might be caused by the fact that *RSEM* estimations tends to report fewer counts for a small number of genes than other abundance estimation tools, as it has been previously discussed in Davidson and Oshlack (2014). This leads to missing true DE genes in downstream analysis, and at the same time to a smaller correlation against the truth datasets.

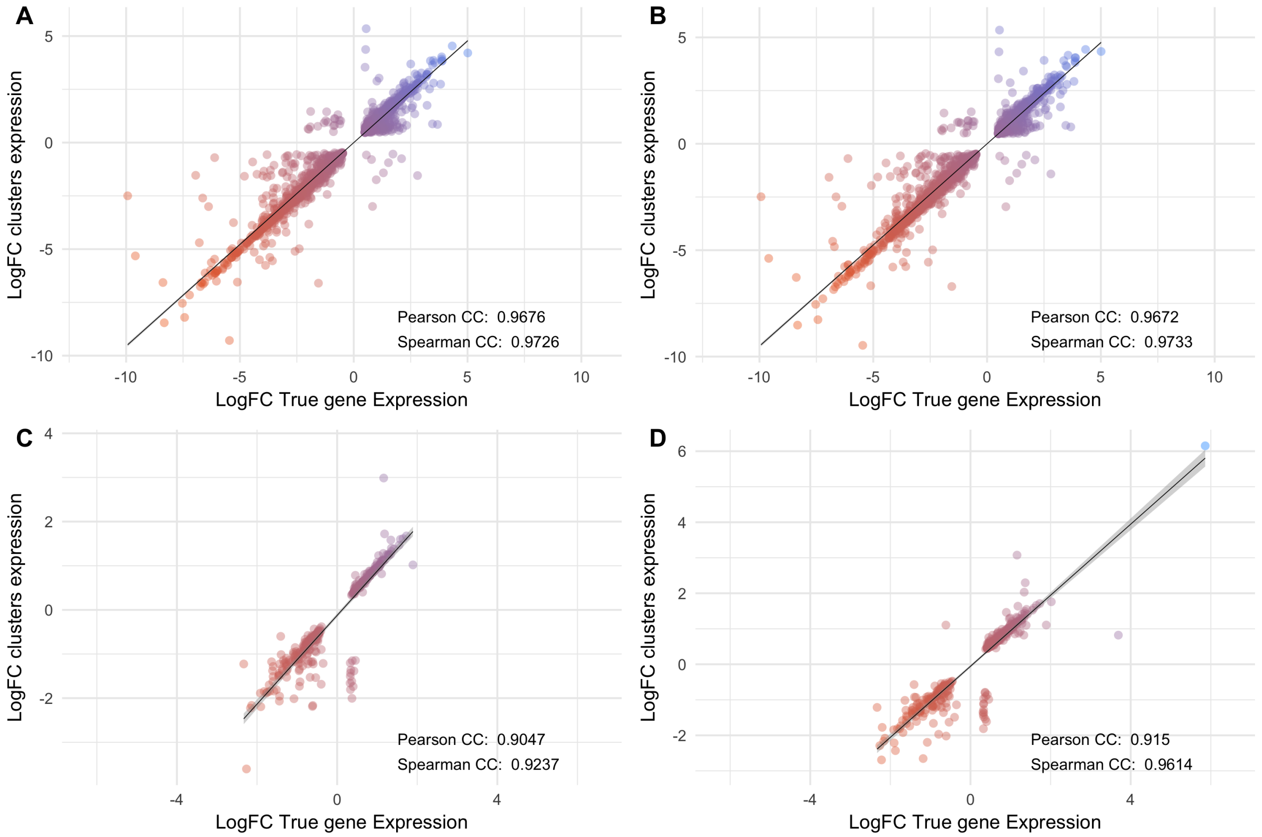

Figure 6. **Effect of contig clustering on differential gene expression analysis.** Significantly differentially expressed clusters were compared to genes tested for differential expression using a genome-based mapping approach. Correlation of log_2 fold changes between significantly differentially expressed clusters compared to genes tested for DE using a genome-based mapping approach for the Arabidopsis and Mouse real datasets. A) and B) show the correlation of log_2 fold change of DE genes of the Arabidopsis real dataset using *RSEM* and *Compacta* for transcript quantification, respectively. C) and D) show the correlation of DE genes of the Mouse real dataset using *RSEM* and *Compacta* for transcript quantification, respectively.

References

Davidson N and Oshlack A (2014) Corset: enabling differential gene expression analysis for de novo assembled transcriptomes. *Genome biology*, 15, 410. doi:10.1186/s13059-014-0410-6.

Frazee A, Jaffe A, Langmead B, and T Leek J (2015) Polyester: Simulating rna-seq datasets with differential transcript expression. *Bioinformatics (Oxford, England)*, 31. doi:10.1093/bioinformatics/btv272.

Frazee AC, Pertea G, Jaffe AE, Langmead B, Salzberg SL, and Leek JT (2014) Flexible isoform-level differential expression analysis with ballgown. *bioRxiv*. doi:10.1101/003665.

G Grabherr M, Haas B, Yassour M, Z Levin J, Thompson D, Amit I, Adiconis X, Fan L, Raychowdhury R, Zeng Q, Chen Z, Mauceli E, Hacohen N, Gnirke A, Rhind N, Di Palma F, Birren B, Nusbaum C, Lindblad-Toh K, and Regev A (2011) Full-length transcriptome assembly from rna-seq data without a reference genome. *Nature biotechnology*, 29, 644–52. doi:10.1038/nbt.1883.

Huber W, Carey VJ, Gentleman R, Anders S, Carlson M, Carvalho BS, Bravo HC, Davis S, Gatto L, Girke T, Gottardo R, Hahne F, Hansen KD, Irizarry RA, Lawrence M, Love MI, MacDonald J, Obenchain V, Ole's AK, Pag'es H, Reyes A, Shannon P, Smyth GK, Tenenbaum D, Waldron L, and Morgan M (2015) Orchestrating high-throughput genomic analysis with Bioconductor. *Nature Methods*, 12, 115–121. doi:10.1038/nmeth.3252. URL `http://www.nature.com/nmeth/journal/v12/n2/full/nmeth.3252.html`.

Kim D, Langmead B, and Salzberg S (2015) Hisat: A fast spliced aligner with low memory requirements. *Nature methods*, 12. doi:10.1038/nmeth.3317.

Li H, Handsaker B, Wysoker A, Fennell T, Ruan J, Homer N, Marth G, Abecasis G, Durbin R, and Subgroup GPDP (2009) The sequence alignment/map format and samtools. *Bioinformatics*, 25, 2078–2079. doi:10.1093/bioinformatics/btp352.

Li W and Godzik A (2006) Cd-hit: a fast program for clustering and comparing large sets of protein or nucleotide sequences. *Bioinformatics (Oxford, England)*, 22, 1658–9. doi:10.1093/bioinformatics/btl158.

Liu H, Ma X, Han HN, Hao YJ, and Zhang XS (2016) Atprmt5 regulates shoot regeneration through mediating histone h4r3 dimethylation on krps and pre-mrna splicing of rkp in arabidopsis. *Molecular Plant*, 9, 1634–164. doi:10.1016/j.molp.2016.10.010.

Malik L, Almodaresi F, and Patro R (2018) Grouper: graph-based clustering and annotation for improved de novo transcriptome analysis. *Bioinformatics*, 34, 3265–3272. doi:10.1093/bioinformatics/bty378. URL `http://dx.doi.org/10.1093/bioinformatics/bty378`.

Patro R, Duggal G, Love MI, Irizarry RA, and Kingsford C (2017) Salmon provides fast and bias-aware quantification of transcript expression. *Nature Methods*, 14. doi:10.1038/nmeth.4197.

Pertea M, Kim D, Pertea GM, Leek JT, and Salzberg SL (2016) Transcript-level expression analysis of rna-seq experiments with hisat, stringtie and ballgown. *Nature protocols*, 11, 1650–67. doi:10.1038/nprot.2016.095.

Pertea M, M Pertea G, Antonescu C, Chang TC, T Mendell J, and Salzberg S (2015) Stringtie enables improved reconstruction of a transcriptome from rna-seq reads. *Nature biotechnology*, 33. doi:10.1038/nbt.3122.

Rhoads A and Au KF (2015) Pacbio sequencing and its applications. *Genomics, proteomics & bioinformatics*, 13, 278–289.

Zhang Z, Schwartz S, Wagner L, and Miller W (2000) A greedy algorithm for aligning dna sequences. *Journal of Computational biology*, 7, 203–214.
